# *Omics* approaches open new horizons in major depressive disorder: from biomarkers to precision medicine

**DOI:** 10.3389/fpsyt.2024.1422939

**Published:** 2024-06-13

**Authors:** Fabiola Stolfi, Hugo Abreu, Riccardo Sinella, Sara Nembrini, Sara Centonze, Virginia Landra, Claudio Brasso, Giuseppe Cappellano, Paola Rocca, Annalisa Chiocchetti

**Affiliations:** ^1^ Department of Health Sciences, Interdisciplinary Research Center of Autoimmune Diseases (IRCAD), Università del Piemonte Orientale, Novara, Italy; ^2^ Center for Translational Research on Autoimmune and Allergic Disease (CAAD), Università del Piemonte Orientale, Novara, Italy; ^3^ Department of Neuroscience “Rita Levi Montalcini”, University of Turin, Turin, Italy

**Keywords:** major depressive disorder, precision medicine, system biomedicine, biomarkers, antidepressant

## Abstract

Major depressive disorder (MDD) is a recurrent episodic mood disorder that represents the third leading cause of disability worldwide. In MDD, several factors can simultaneously contribute to its development, which complicates its diagnosis. According to practical guidelines, antidepressants are the first-line treatment for moderate to severe major depressive episodes. Traditional treatment strategies often follow a one-size-fits-all approach, resulting in suboptimal outcomes for many patients who fail to experience a response or recovery and develop the so-called “therapy-resistant depression”. The high biological and clinical inter-variability within patients and the lack of robust biomarkers hinder the finding of specific therapeutic targets, contributing to the high treatment failure rates. In this frame, precision medicine, a paradigm that tailors medical interventions to individual characteristics, would help allocate the most adequate and effective treatment for each patient while minimizing its side effects. In particular, multi-omic studies may unveil the intricate interplays between genetic predispositions and exposure to environmental factors through the study of epigenomics, transcriptomics, proteomics, metabolomics, gut microbiomics, and immunomics. The integration of the flow of multi-omic information into molecular pathways may produce better outcomes than the current psychopharmacological approach, which targets singular molecular factors mainly related to the monoamine systems, disregarding the complex network of our organism. The concept of system biomedicine involves the integration and analysis of enormous datasets generated with different technologies, creating a “patient fingerprint”, which defines the underlying biological mechanisms of every patient. This review, centered on precision medicine, explores the integration of multi-omic approaches as clinical tools for prediction in MDD at a single-patient level. It investigates how combining the existing technologies used for diagnostic, stratification, prognostic, and treatment-response biomarkers discovery with artificial intelligence can improve the assessment and treatment of MDD.

## 1 Major depressive disorder

Major depressive disorder (MDD) is a common, heterogenous, episodic, and disabling psychopathological condition characterized by a combination of signs and symptoms that negatively impact patients’ productivity and well-being, including cognitive dysfunction, emotional regulation, motor activity, motivation, and possible suicidal ideation (1, 2). MDD is the most prevalent psychiatric condition, represents approximately 30% of mental disorders, and affects more than 300 million people worldwide, with 5–17% of the world population suffering from the disorder at least once in their lifetime (3). In 2019, MDD was the third cause of years lived with disability in the world’s adult population and the World Health Organization (WHO, Geneva, Switzerland) estimates that, by 2030, depression will have become the leading cause of disability worldwide (4–7). Moreover, MDD is associated with high levels of morbidity and mortality: patients often develop comorbid mental conditions, including posttraumatic stress disorder, anxiety disorders, obsessive-compulsive disorder, substance use disorders, and a lifetime risk of a suicide attempt of 31% (8, 9). The disorder is also associated with non-psychiatric chronic illnesses, including cardiovascular and cerebrovascular diseases, metabolic disorders like type 2 diabetes mellitus and obesity, and cancer (9). In relation to the abovementioned comorbidities, patients’ life expectancy is reduced by 7–13 years (10). In addition to the impact on the people affected, there are economic and social implications, such as the reduction in productivity, higher healthcare costs, and costs incurred by unpaid caregivers (11, 12).

The diagnosis of MDD is based on the assessment of symptoms by the clinician, the self-assessed disease features, and the patient’s lifetime history (13). The International Classification of Diseases (ICD) (from the 6th to the 11th edition) and the Diagnostic and Statistical Manual (DSM) (from I to V edition) provide a set of criteria for diagnosing a major depressive episode (MDE). According to the DSM-5 (14), the simultaneous manifestation of at least five of the nine following groups of symptoms over a two-week period is needed: depressed mood; markedly diminished interest or pleasure in activities; reduced ability to think or concentrate, or indecisiveness; feelings of worthlessness, or excessive or inappropriate guilt; recurrent thoughts of death, or suicidal ideation, or suicide attempts or plans; insomnia or hypersomnia; significant change in appetite or weight; psychomotor agitation or retardation; and fatigue or loss of energy (14). Of these five symptoms, one of the first two must be present (14). A diagnosis of MDD can be made if an MDE is present or occurred in the patient’s history and is not explained by a concomitant primary psychosis or by a known bipolar disorder (14). Indeed, the main differential diagnosis to evaluate is bipolar disorder (BD), with approximately 40% of patients with BD initially misdiagnosed with MDD (15). Therefore, all patients showing symptoms of depression should be screened for BD (16).

The binary approach proposed in the DSM and ICD defines MDD as absent or present. This dichotomic classification has some limitations as it represents a *post-hoc* construct that reduces information, precision, validity, and reliability of the MDD diagnosis (13). Further characterization of the disorder is needed to overcome the problem of binary diagnosis. This should include an assessment of the current depressive symptoms and suicidal behavior as well as a detailed history of the disorder, including the number, duration, and severity of relapses, and the response to treatments coupled with a good knowledge of the patient’s life history, useful to differentiate between physiological and pathological human stress-related responses (13, 16). The severity of symptoms can be measured with standardized psychometric scales (3) such as the Hamilton Depression Rating Scale (17), Montgomery-Asberg Depression Rating Scale (18), and Beck Depression Inventory (19). Suicidality, in terms of suicidal ideation and attempts, is among the stronger predictors of suicide (20) and should be assessed with validated scales like the Columbia-Suicide Severity Rating Scale (21).

The clinical characterization of MDD is needed not only for diagnostic purposes but also to develop a personalized management plan at a single-patient level (16). This profiling consists of assessing various clinical domains, including clinical subtypes, neurocognition, clinical staging, and functioning and quality of life. MDD is considered a heterogeneous disorder, and many clinical subtypes with specific associations of signs and symptoms were described, including melancholic, atypical, anhedonic, inflammatory, suicidal, anxious, somatic-traits, reactive, psychotic, pseudo-demented, and seasonal subtypes (3, 16). A worse prognosis and a specific treatment indication consisting of antidepressants and antipsychotics were supported by sufficient evidence only for the psychotic subtype that includes delusions and/or hallucinations in the clinical context of a severe MDE (16), suggesting that this specific subtype may be studied as a partial autonomous nosological entity.

Neurocognitive impairment is present in 85–94% of cases during an MDE and 39–44% of cases during remissions. It mainly involves attention, short-term memory, and executive function and accounts for a disproportionately high percentage of patients who have not completely reached the psychosocial functioning they used to have before the onset of the MDE (22–24). A better identification and characterization of patients with MDD with neurocognitive dysfunction might promote the development of new and personalized treatments (25).

Clinical staging refers to two main classifications: the first one is related to the course of the disorder and the second one to the response to treatment (26, 27). The former defines the following five stages: (i) a prodromal phase with (a) non-specific significant or (b) subthreshold depressive symptoms; (ii) a second stage corresponding to the first MDE; (iii) a residual phase characterized by (a) non-specific, (b) residual depressive, or (c) mild chronic depressive (dysthymia) symptoms; (iv) a fourth stage corresponding to (a) a recurrent MDE in a patient that fully recovered from a previous depressive episode or (b) double depression, i.e., an MDE superimposed on dysthymia; and (v) a persistent MDE lasting at least two years without interruption (26). The second classification also defines five stages: (0) no history of treatment failure; (1) failure of one adequate treatment, i.e., 6–8 weeks for antidepressants or 36–52 weeks for psychotherapy; (2) failure of two adequate treatments (12–16 weeks for antidepressants or 36–52 weeks for psychotherapy); (3) failure of three adequate treatments; (4) failure of three or more adequate treatments with at least one involving augmentation or combination strategies (27). Stage 2 corresponds to the most common definition of treatment-resistant depression (TRD) and stage 4 to refractory depression (28).

According to the DSM-5 and ICD-11, an MDE results in a significant impairment in personal, family, social, educational, occupational, or other important areas of functioning (29). In parallel, patients report an important reduction in their self-rated quality of life and satisfaction with life and expect from treatments a restoration of positive emotions, functioning, and meaningfulness of life rather than merely symptom relief (30, 31). The routine assessment of functioning, quality of life, and life satisfaction with standardized instruments like the recovery index promotes a patient-centered perspective, shifting the focus from a symptomatic remission to a functional and personal recovery from MDD (16, 32).

An in-depth clinical characterization can help clinicians in treatment planning and potentially identify more homogenous phenotypes of MDD that may be related to specific biological processes, thus partly overcoming the obstacle of the clinical and biological extreme heterogeneity of this psychopathological condition.

About pathogenesis, it is known that MDD emerges as a result of a complex and mostly obscure interplay between genetic vulnerabilities and environmental factors. There is evidence that female sex, family history, childhood maltreatment, as well as more recent stressors, are risk factors for the development of the disorder. However, a clear understanding of how genetic information interacts with early and recent environmental exposure through epigenomic mechanisms is not available (9). At a genetic level, no single variation was found to be solely responsible for an increased risk of MDD development (33); instead, a combination of variations in over 100 gene loci confer a genetic susceptibility towards disease incidence. Even though MDD hereditability traits can range between 30–50%, social and environmental factors, such as stress and the occurrence of traumatic events, can greatly impact mental health and drive disease pathogenesis (34). As an example, the recent COVID-19 pandemic has led to an increased prevalence of MDD, not only in COVID-19 patients but also in relatives and significant persons, such as close friends (35). Other hypotheses that justify the diversified etiology of MDD have identified dysregulated biochemical pathways, including hormone dysregulation and glucocorticoid increase, monoamine neurotransmitter deficiency (serotonin, dopamine, and norepinephrine), exacerbated neuroinflammation and scarcity of glial cells in the brain (36–38). Postmortem studies of patients with MDD have reported a neuroprogression associated with the disorder consisting of changes in the density and size of neurons and glia in several brain regions combined with a reduced expression of synaptic genes (39).

Despite a wide range of effective treatments, including antidepressants, evidence-based psychotherapies, nonpharmacological somatic treatments, and numerous augmentation strategies, about half of patients remain nonresponsive or poorly responsive to available treatments, thus failing to reach symptomatic remission and functional and personal recovery (9, 40).

Precision medicine, which integrates clinical and biological parameters specific to each individual to stratify treatment groups, may represent a solution to the current standard trial-and-error approach to treatment, moving to a more tailored method that might improve the prognosis of MDD through the allocation of the best treatment while minimizing side effects.

## 2 Precision medicine: a patient-tailored approach

The multifactorial etiology of MDD, as well as the numerous available treatments, mostly administered on a trial-and-error basis, portray MDD as an interesting target for the application of precision medicine (41). Also described as personalized medicine, this approach aims to combine biologic high-throughput data generated through *omic* approaches and environmental and lifestyle factors to identify specific individual features predictive of disease susceptibility, prognosis, and treatment response (42). The idea of applying a personalized treatment in psychiatry has been present for more than half a century (43), but only in recent years it became possible to generate and interpret very large datasets, called “big data”, and obtain complete individual profiling able to provide patient-specific details that can be used as diagnostic, prognostic, and treatment response biomarkers or therapeutic targets (44). In fact, constant technological advances led to the shift from basic blotting techniques (Northern, Southern and Western blotting) (45) to advanced genome-wide associating studies (GWAS) (46), single-cell transcriptomics (47) and imaging mass cytometry (48), generating larger amounts of data, which can be nowadays analyzed through artificial intelligence (AI) and machine learning techniques (49).

This opposes the classical approach or “imprecision medicine”, as labeled by Schork, that has led to a huge economic burden associated with MDD: in 2015, the fifth highest-grossing drug in the United States was duloxetine, a serotonin and norepinephrine reuptake inhibitor used to treat depression (50), which was only able to help one in every nine patients (51). Indeed, MDD response heterogeneity remains a critical problem for developing algorithms for implementing recovery rates in clinical practice (52). Inevitably, this represents a significant economic weight for patients and national health systems, incentivizing public and private investment in precision medicine (53).

Various techniques including magnetic resonance imaging (MRI), positron emission tomography (PET) scans and electroencephalography (EEG), have been adopted in the context of MDD, as reviewed in (54); also, as previously introduced, several *omic* approaches, most commonly genomic (55), transcriptomic (56), proteomic (57), and metabolomic (58) studies have been explored, but a definitive standardized and efficient strategy to accurately categorize patients and apply the appropriate therapy remains elusive.

*Genomics* aims to find genetic variants associated with disease, response to treatment, or patient prognosis. In this regard, the principal technologies used are whole-genome or whole-exome sequencing, targeted next-generation sequencing (NGS), genotyping and GWAS, which allow for the identification of thousands of genetic variants associated with complex diseases (59, 60).

*Epigenomics* examines reversible modifications of DNA or DNA-associated proteins, such as DNA methylation or histone acetylation, that can be influenced by both genetic and environmental factors (59, 60). These modifications are frequently detected in pathological conditions, including MDD (61). The technologies used to evaluate epigenetic modifications encompass bisulfite treatment of DNA before routine NGS to evaluate DNA methylation, immunoprecipitation and NGS-based analysis to assess the protein-DNA interaction, and ATAC-seq (Assay for Transposase-Accessible Chromatin using sequencing) to estimate genome-wide chromatin accessibility (59, 60).

*Transcriptomics* focuses on RNA transcripts analyzing expression levels of both quantity and quality of the transcripts, including messenger RNA (mRNA) and non-coding RNA (ncRNA). Transcriptomics can be performed using both bulk and single-cell methods. Common techniques used in bulk transcriptomics are microarrays and RNA sequencing (RNA-seq); among single cell analysis the gold standards are targeted single-cell transcriptomics (using for example, BD Rhapsody) and scRNA-seq (Smart-Seq2) (59, 60).

*Proteomics*, on the other hand, focuses on the study of peptide abundance, modification, and interactions. The main technologies used are mass spectrometry (MS)-based, which ensure high-throughput analyses of thousands of proteins in cells or body fluids. Additionally, flow cytometry, including advanced technologies such as FACS (Fluorescence-Activated Cell Sorting) and CyTOF (Cytometry by Time of Flight), and fluorescence imaging techniques are used to evaluate extra- or intracellular proteins, providing information for a large number of cells at a relatively low cost (59, 62).

*Metabolomics* quantifies various types of small molecules that reflect metabolic functions, in order to measure the response to perturbations like those connected to disease, as well as to observe the intricate relationship between physiology and external events (59). Techniques like liquid chromatography-MS (LC-MS) are appropriate for human cohort discovery investigations since they can accurately monitor tens to hundreds of metabolites simultaneously (63).

*Microbiomics* involves the study of an entire microbial community, identifying and quantifying the molecules that contribute to its structure, dynamics, and function (59, 64). In particular, *metagenomics* is the comprehensive analysis of uncultured microorganisms and hosts’ genetic material that applies genomic technologies, like metagenomic NGS (mNGS), in patients’ samples. It represents a significant upgrade from classical molecular assays that target only a restricted number of microbes and give a limited description of the pathogens through the diversity of one gene, using for instance the 16S ribosomal RNA (rRNA) gene. The study of RNA and proteins from microbiota can also be implemented through *metatranscriptomics* or *metaproteomics* (65, 66).

In the specific case of brain disorders, *connectomics* utilizes neuroimaging techniques, mainly MRI, to provide an exhaustive map of the whole neural connections, representing the study of functional and structural brain connectivity (67, 68). These techniques can improve the identification of personalized targets for neuromodulatory treatments (69).

All the above-mentioned *omic* technologies may help to identify new depression-specific biomarkers, even though this may rely on the lack of integration of such big data by system biomedicine approaches (41, 70). Overall, understanding the complex interplay of genetic, environmental, and physiological factors in MDD is crucial for improving prevention and treatment strategies for this debilitating condition. For that, an integrated multi-*omic* approach, which not only combines data from the single different *omics* but also considers the interactions between them, is gaining importance as an innovative strategy for biomarker discovery and validation in MDD (46).

In particular, *cytomics* is a multi-*omic* strategy used to determine the molecular phenotype of single cells that links various aforementioned omics sciences with cell and tissue dynamics and function, considering also its modulation by external factors. Naturally, it takes advantage of techniques that are transversal to other *omic* sciences, such as flow cytometry and fluorescence microscopy, to generate a wide array of data from each single cell of interest (71).

Lastly, *immunomics* integrates molecular immunology, genomics, proteomics, transcriptomics, cytomics and bioinformatics by studying antigens or epitopes that interact with the host’s immune system with a multi-layer *omic* approach (72, 73). There has been increasing evidence that MDD is linked to a systemic immunological activation over the last two decades (74), affecting inflammatory markers, immune cell counts, and antibody titers (75, 76).

**Figure 1:**
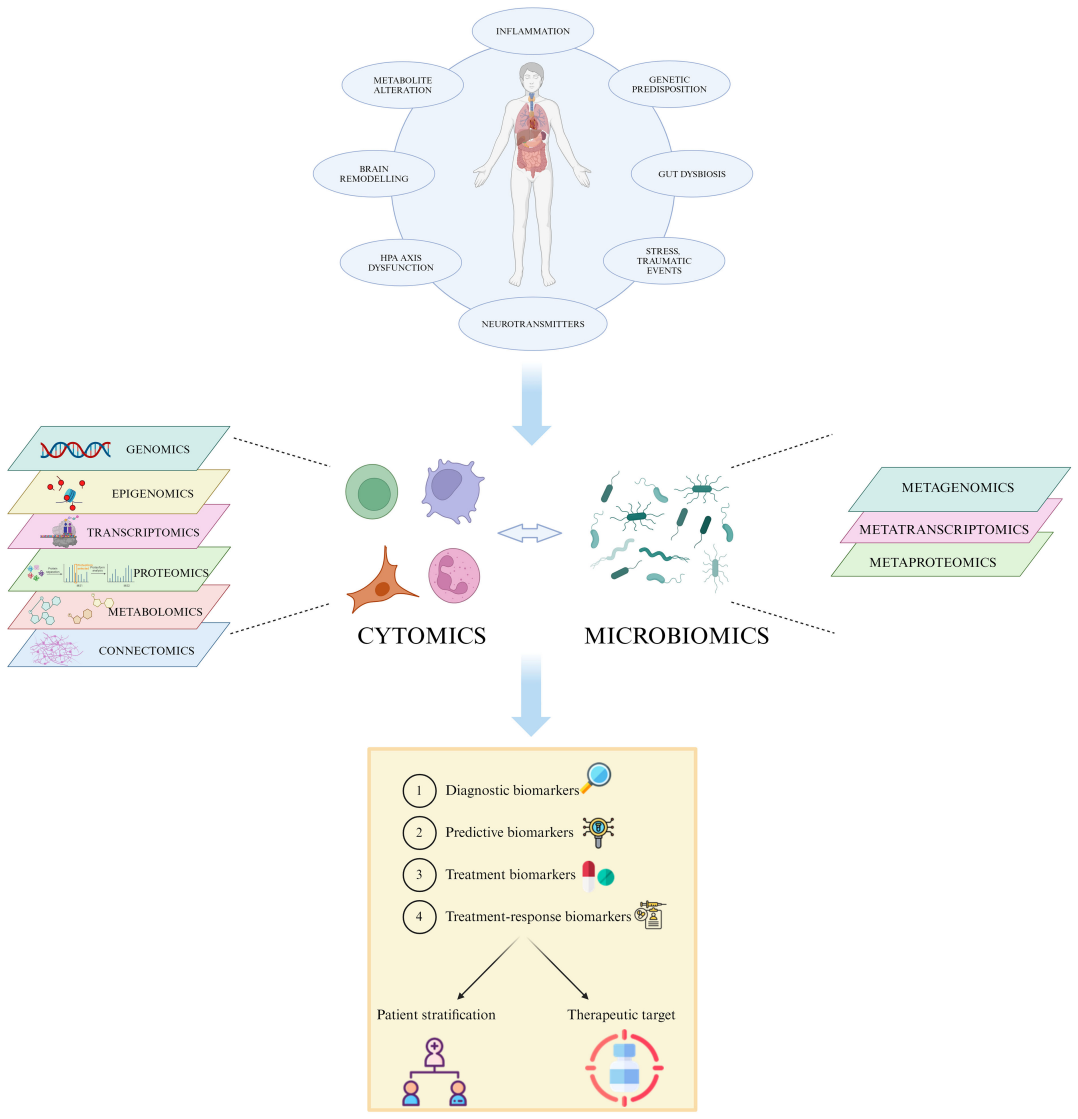
Multi-*omic* approach in MDD. The heterogeneity of MDD and its diverse etiology hinders the accurate patient stratification and appropriate treatment choice. The integration of high-throughput data generated by single *omic* technologies, such as genomics, transcriptomics and proteomics, into a multi-*omic* system permits the identification of specific individual features able to predict disease susceptibility, prognosis, and treatment response, leading to a more effective personalized care. Created with BioRender.com.

In this review, the promising role of precision medicine-based approaches as diagnostic, prognostic and therapeutic tools for MDD will be explored, focusing particularly on the potential of biomarkers associated with the immune system as well as the current state of AI and machine learning approaches applied to the psychiatric field (**Figure 1**).

## 3 Application of precision medicine in MDD

Given the challenges in accurately diagnosing and treating MDD, which has a misdiagnosis rate of approximately 66% (77) and a treatment-resistant prevalence of 31% (78), finding more effective alternative approaches has become essential. By integrating both biological and non-biological characteristics of individuals, precision medicine facilitates the clustering of patients, thereby enabling more tailored and effective treatment strategies to improve diagnosis, treatment, and prevention efficacy (77, 79).

Precision medicine, with its multi-*omic* profiling that integrates genetic and epigenetic data, biomarkers, clinical characterization, and environmental exposures, holds revolutionary potential in psychiatry, aiming to enhance psychiatric diagnoses and treatment efficacy (77, 79). Through the analysis of multi-*omic* signatures, researchers can gain a deeper understanding of the complex interplay between genetic predispositions, metabolic pathways, and environmental influences in the onset and progression of depression, ultimately leading to more effective personalized treatment strategies for MDD (46, 77, 79). Also, another perceived challenge to adopt the biomarker approach in depression and other psychiatric disorders has been the difficulty in accessing tissue samples, a hurdle that has been surmounted in diseases like cancer, where most advances in precision medicine have occurred (80). However, the recent use of circulating cancer biomarkers, such as microRNAs (miRNAs), to detect early metastatic spread has validated the potential of liquid biopsy. This is highly relevant to molecular studies in MDD, where changes in circulating miRNAs, regulatory enzymes, and inflammatory cytokines have been linked to treatment outcomes (81).

Biomarkers are defined as “indicators of normal biological processes, pathogenic processes or pharmacological responses to a therapeutic intervention that can be measured and evaluated objectively” (82). In clinical practice they can be distinguished in: i) diagnostic biomarkers, if they are able to discriminate between the presence or absence of a specific disease, for example supporting the differential diagnosis between MDD and BD in patients with a first MDE; ii) predictive biomarkers, if they can predict the disease onset; iii) treatment biomarkers, if they can predict optimal treatment options and iv) treatment-response biomarkers, if they can measure the effectiveness of the treatment (83).

Given that MDD is linked not only with changes in brain structure and function, but also with gastrointestinal factors, the immune system, the endocrine system, neurotrophic factors, hormones, and oxidative stress, there are a plethora of biomarkers associated with these domains. Unfortunately, due to the heterogeneity of the disease, no specific biomarker has been found for MDD yet (84).

### 3.1 Soluble biomarkers – proteomics and metabolomics in MDD

Currently, a wide range of soluble biomarkers are used in the context of MDD, including endocrine hormones, monoamine neurotransmitters, neuropeptides, immune and inflammatory products, growth factors, and metabolic substances (such as adipokines and lipidemic factors). Among them, those linked to the enhanced activity of the Hypothalamic–pituitary–adrenal (HPA) axis, such as cortisol, serotonin, and brain-derived neurotrophic factor (BDNF), are the most studied for MDD (85). However, finding a more specific biomarker or a panel of biomarkers able to reflect the patients’ neuroinflammatory state and response to therapy has become a pressing need. In this context, immune and inflammatory markers such as cytokines and oxidative stress elements have been extensively studied in association with depression. Indeed, several studies and meta-analyses highlighted a strong association between inflammation and MDD, revealing elevated concentrations of C-reactive protein (CRP), interleukin-6 (IL-6), IL-12, tumor necrosis factor-α (TNF-α), and IL-1β in the serum of MDD patients (81, 86). Interestingly, the increased levels of some of these cytokines (i.e. IL-6, TNF-α, CRP and IL-1β) have been reported to correlate with lower response to pharmacological treatment (87). These findings are in line with a recent work by Xu et al., showing that specific sets of peripheral cytokines reached a good accuracy in discriminating non-responders from responders with sensitivity and specificity over 80%, revealing them as promising biomarkers for MDD diagnosis and antidepressant response (88). Along with inflammation, a recent study by Ait Tayeb et al. suggests that also oxidative stress, measured on various matrices such as serum, plasma, or erythrocytes, has a critical role in MDD according to disease stage and clinical features (89).

In recent years, approaches that integrate multi-*omic* platforms have been explored with the purpose of accelerating clinical biomarker discovery and deeply investigating the pathophysiological mechanisms in psychiatric disorders. Among them, MS-based proteomic techniques can analyze the whole proteome of an individual in an unbiased manner, overcoming the limitations encountered with more traditional methods such as western blotting or enzyme-linked immunosorbent assay (ELISA), allowing for the discovery of novel disease biomarkers. In a work by Silva-Costa et al., an untargeted MS proteomic approach unveiled a first potential biomolecular signature for late life depression and the biological pathways related to this condition, which could be suitable targets for the development of novel strategies for its prevention, early diagnosis, and treatment (90). In another work by Schubert and colleagues, a workflow was developed starting from the phenotype of cognitive impairment in remitted MDD to illustrate an application of systems biology approach; using a weighted gene co-expression network analysis (WGCNA) in the discovery phase, followed by a targeted proteomic analysis of the results, the authors were able to identify cellular mechanisms and candidate biomarkers for cognitive dysfunction in MDD (91).

On the other hand, metabolomics can provide a functional readout of the inner cell state and help identify biochemical signatures or biomarkers specific to different disorders, including MDD (92). In particular, MS-based analytical metabolomic approaches can be used in the study of psychiatric conditions due to their high-throughput capacity and ability to resolve a large number of metabolites with little to no need for purification (93). In a work conducted by Pan et al., using a GC-MS coupled with LC-MS/MS-based targeted metabolomics approach in the early stage of MDD, the authors identified a plasma neuro-metabolite signature able to discriminate first-episode, antidepressant drug-naïve depressed patients from healthy controls with high accuracy. Notably, their results indicate that the identified biomarker panel – composed by gamma-aminobutyric acid (GABA), dopamine, tyramine and kynurenine – was able to accurately diagnose blinded samples with both high sensitivity and high specificity (94). MacDonald et al. consider metabolomics a cost-effective and non-invasive technique to screen MDD individuals, monitoring their response to treatment and guiding their management. Additionally, combined with the screening of online drug registries, metabolomics can be a helpful tool to accelerate the discovery of novel druggable targets (92).

### 3.2 Genetic biomarkers – genomics and transcriptomics in MDD

Genomics, by finding genes that contribute in predicting MDD susceptibility and therapy response, has played a significant role in precision medicine for MDD, expanding the field of pharmacogenetics and increasing clinical outcomes through an accurate diagnosis (95–97). Genetic predisposition in MDD relies on a polygenic trait: approximately 40% to 70% of MDD patients have susceptibility loci mainly located in genes involved in the serotonergic system and in the HPA axis, such as polymorphisms in 5-hydroxytryptamine (5HT; serotonin) transporter (*5-HTT*) and in tryptophan hydroxylase (TPH) genes (*TPH1* and *TPH2*) (95). However, other genes have been studied in association with antidepressant response and thus they could be considered as treatment-response genetic biomarkers for MDD. This group includes catechol-O-methyltransferase (*COMT*) which plays a central role in noradrenaline degradation (98, 99), monoamino oxydase A (*MAOA*), that contributes to monoamines degradation (100–102), multi-drug resistance 1 (*MDR1/ABCB1*) genes encoding P-glycoprotein (103–105), glutamate receptor ionotropic kainate 4 (*GRIK4*) (106, 107), Potassium Two Pore Domain Channel Subfamily K Member 2 (*KCNK2/TREK1)*, which encodes a neuronal potassium channel, Phosphodiesterase 2A (*PDE2A)* encoding for the enzyme which metabolizes cyclic AMP, transcription factor cyclic adenosine monophosphate response element binding protein (*CREB1*), *BCL2* and many other candidate genes (108, 109).

In order to advance the understanding of the complex genetic architecture of depression and provide new tools for further investigating the disease etiology, several studies of genome-wide meta-analysis have been performed. Among them, a large-scale genome-wide association study conducted by Howard and colleagues identified 102 independent variants, 269 genes, and 15 gene sets associated with depression, including both genes and gene pathways linked to synaptic structure and neurotransmission (55). Interestingly, among the identified genes, the ones implicated in immune dysregulation have been extensively discussed in (110). In particular, Tubbs and coworkers grouped these 34 immune-related genes in 5 main categories based on their functions. Into the first category falls the majority of these genes, which contributes to general lymphoid development, implicating multiple white blood cell types. In contrast, other genes exhibit cell-type specific functions, for instance, some genes are involved in T-cell activation and development, other are crucial regulators of B cells, some of them have been associated to antigen presentation or are cytokine-related genes. Finally, some played a role in neuroinflammation, highlighting again the close link between immune system and brain functions (110).

Of note, among the predictive markers of treatment-response, in the context of personalized medicine, one of the most interesting categories is represented by the drug-metabolizer enzymes belonging to cytochrome P450 (CYP) family, which comprise more than 200 isoenzymes responsible for antidepressant drugs’ metabolism (111). Indeed, several single nucleotide polymorphisms (SNPs) in CYP family genes may significantly impact the metabolism of these drugs, resulting in different categories of patient metabolizers: poor metabolizers, intermediate metabolizers, extensive metabolizers, and ultra-rapid metabolizers. This is crucial when considering the potential for adverse effects or therapy inefficacy (112–114). However, the CYP genotyping is still not used in clinical application to predict MDD treatment response. Thus, integrating pharmacogenetic testing for CYP enzymes into MDD clinical management could allow healthcare providers to establish a personalized antidepressant treatment, which would minimize the risk of adverse drug reactions and improve the effectiveness of pharmacotherapy for depression (112–114).

In recent years, also miRNAs have emerged as attractive clinical biomarkers for diagnosing depression and other psychiatric disorders. Indeed, miRNA levels have been found impaired in schizophrenia, bipolar disorder, and depression (115). In particular, it has been demonstrated that polymorphisms in the let7 miRNA family and a variant of the miR-30 family (specifically miR-30e) lead to increased MDD susceptibility (116, 117). More specifically, let-7c and let-7b miRNA expression have been found significantly lower in treatment-resistant patients (118). Interestingly, a bioinformatic analysis revealed that those miRNAs are involved in the regulation of PI3K-AKT-mTOR signaling pathway, which has been previously reported to be dysfunctional in depression (119). Circulating miR-134 is another example of biomarker for neuropsychiatric disorders, reaching 79% sensitivity and 84% specificity in MDD patients. Furthermore, reduced levels of miR-200 have been found in depressed individuals, while the miR-144 and miR-146a have an inverse relationship with depressive symptoms (85). However, the interpretation of differential gene expression in brain tissue homogenates remains challenging due to the heterogeneous cellular composition of the sample. Thus, in the last years, there has been a rising interest in using single-cell sequencing approaches, which have revealed that gene expression patterns in the brain are cell-type specific. A work by Nagy et al. uses a single-nucleus transcriptomic technique in cells from the dorsolateral prefrontal cortex of patients with MDD that committed suicide, revealing the dysregulation of gene expression in almost 60% of the cell types identified, with a total of 96 differentially expressed genes. Given the complexity of psychiatric disorders such as MDD, investigating the role of each cell subtype in the brain could be relevant and requires single-cell resolution techniques (120). A similar approach has been used in non-human primates to integrate single-nucleus RNA-sequencing (snRNA-seq) and spatial transcriptomics: the snRNA-seq data resulted in the identification of six enriched gene modules linked to depressive-like behaviors, which were resolved by spatial transcriptomics. Findings indicate cell-type and cortical layer-specific gene expression changes and identify one microglia subpopulation associated with depressive-like behaviors in female non-human primates (121). This evidence indicates that combining these two approaches of snRNA-seq and spatial transcriptomics allows to improve spatial resolution of depression traits, highlighting the role of microglia in stress-inflammation.

### 3.3 Immune system – cytomics and immunomics in MDD

There has been increasing evidence that MDD is linked to a systemic immunological activation over the last two decades (74), affecting inflammatory markers, immune cell counts, and antibody titers (75, 76). Therefore, inflammation is a crucial factor to consider when studying MDD (122): it has already been reported that inflammatory diseases, e.g. rheumatoid arthritis, can increase the risk of developing depressive symptoms (123); not only that, levels of pro-inflammatory molecules are often augmented in MDD patients (124), and the administration of antidepressants together with anti-inflammatory drugs, such as celecoxib, are able to counteract more quickly depressive episodes (125). However, this strategy should be considered with caution, considering that anti-inflammatory treatment is highly unspecific and only half of MDD patients have a notable inflammatory profile (126).

The immune dysregulation extends beyond peripheral organs, also impacting the brain (127). Interestingly, similar immune system changes have been found in other mental health conditions like bipolar disorder and schizophrenia. This suggests that there might be common pathways in all these conditions (87). For example, after persistent inflammatory stimuli, many cell types of the brain undergo morphological, functional, and quantitative changes, in particular microglia and astrocytes (128). Unexpectedly, evidence also shows the opposite: some patients are affected not only by immune activation but also by immune suppression (129). This further complicates the immunological picture of MDD, which may thus reflect the effects of an infection, an autoimmune disorder, or genetic sensitivity that can exacerbate the innate and adaptive immune systems’ response to stress. For instance, autoimmune disorders and infections can influence CD4^+^ T helper (Th) cell function, cytokine, and antibody production (74).

In the central nervous system (CNS), cytokines are produced and released by immune (such as microglia), endothelial or neuronal cells, being key regulators of inflammation and cellular activities, and playing a crucial role in MDD pathology (130). There are primarily two types of cytokines: pro-inflammatory cytokines, which promote inflammation, and anti-inflammatory cytokines, which mitigate it (131). In the brain, cytokines are involved in organ development, influencing neuronal integrity, neurogenesis, and synaptic remodeling (132). However, psychological and physical stressors may prolong the activation of pro-inflammatory cytokines to a pathological level, disturbing multiple neuronal functions, like neurotransmitter impairment, apoptosis, and reduced neurogenesis (133, 134), thus supporting MDD-related neuroprogression (135).

Circulatory cytokines, instead, can impact brain inflammation through various pathways, including humoral (through leaky regions of the blood-brain barrier (BBB)), neural (through signals via afferent nerve fibers, in particular vagus nerve), and cellular routes (through stimulation of microglia in order to attract monocytes in the brain), crossing the BBB (136, 137). In particular, several pathways can lead to immune/cytokine dysfunction that contribute to the pathogenesis of depression: first, an activation of HPA due to environmental stress, as well as elevated systemic pro-inflammatory cytokines, leads to an increase of cortisol production. Even though cortisol is typically immunosuppressive, emerging theories suggest that elevated levels may lead to glucocorticoid resistance in immune cells, interrupting the inhibitory feedback mechanism. Additionally, cortisol is proposed to have a pro-inflammatory activity during stress by stimulating the extrahepatic enzyme 2,3-indolinime dioxygenase (IDO), which it is found in different types of cells including brain and immune cells (138, 139), leading to serotonin depletion. Specifically, IDO breaks tryptophan, a precursor of serotonin, into kynurenine, leading again to a serotonin depletion (140). Kynurenic acid, then, is not neurotoxic itself, but when it is metabolized to quinolinic acid (141) it modulates the synthesis of IL-1β and IL-6. These cytokines inhibit the glutamate re-uptake, therefore impairing the production of trophic factors while decreasing the brain plasticity and increasing the oxidative stress damage (142). Consequently, significant tissue damage occurs in many brain regions implicated in mood regulation (143) which further triggers the inflammatory response (144). This explains the detection of neuroactive substances such as quinolinic acid in the plasma and cerebrospinal fluid of patients with MDD (132).

The inflammatory process in MDD is influenced by stress-induced danger/damage-associated molecular patterns (DAMPs, also known as alarmins) involving nuclear factor kappa B (NF-κB) and the inflammasome pathway (74). It is important to distinguish between inflammation and para-inflammation, as both conditions coexist in MDD. The former is triggered by pathogens via pathogen-associated molecular patterns (PAMPs), while the latter is induced by psychological stress or DAMPs, and therefore it is defined as “sterile inflammation”. DAMPs, produced in response to stress, activate innate immune cells via the toll-like receptor (TLR) pathway, in particular TLR4, stimulating pro-inflammatory cytokine production, such as IL-1β, TNF-α, and IL-18. These cytokines have been found with a higher expression in patients with MDD, suggesting their involvement in the disease (145, 146). Notably, as evidence of the significance of TLR4 involvement in MDD, its blood level decreases in patients following a successful treatment (147).

Given the relevance of inflammation and the abundance of pro-inflammatory biomarkers in MDD, understanding the role of immune cells becomes of paramount importance, as these are able to produce but also get modulated by cytokines (148). In a physiological state, an effective network of mononuclear phagocytes is present in the CNS. The most prevalent cells in this network, around 10% of cells in the brain, are called microglia and they are considered the CNS’s innate immune system. They are produced as early myeloid progenitors in the embryonic yolk sac and move to populate the entire parenchyma (149). Peripheral immune cells, on the other hand, cannot enter the parenchyma, being only present in the meningeal borders of the CNS, as the presence of adaptive immunity within the brain parenchyma is symptomatic of chronic brain infection, inflammation or autoimmunity (150, 151).

The high interaction between microglia and neurons plays a fundamental role in shaping brain circuits by influencing the intensity of synaptic transmissions and modelling neuronal synapses. These interactions have additional tasks, such as phagocytosing and removing bacteria, dead cells, protein aggregates, and other particulate and soluble antigens that could harm the CNS after injury (149, 152). They contribute to various aspects of immune responses and tissue repair in the CNS, comprising the secretion of soluble factors, such as cytokines or neurotropic factors (153). Microglia continuously monitor their surrounding with motile multiple branches and processes to survey any disturbance. When they become amoeboid-shaped it indicates a highly activated state linked to pro-inflammatory processes, mirroring their reaction to a wide range of stimuli (154).

Their activation is categorized as classical (M1) or alternative (M2). M1 activation leads to a pro-inflammatory and neurotoxic state; M2 activation, on the contrary, promotes the release of anti-inflammatory cytokines (155). Disrupting the balance between these two activation states through neuroinflammation can lead to impaired microglial functions, which in turn have the potential to promote acute or chronic pathologic processes in the CNS (149). For example, abnormal activation of microglial cells was noticed in patients with MDD, along with a decrease in neurogenesis and an increase in glutamate toxicity (156–158).

Several studies have evaluated the immunophenotypic changes in peripheral blood immune cells, recently summarized and meta-analyzed in two systematic reviews (148, 159). Although in both cases the authors conclude that immune cell alterations can be found in MDD patients when compared to controls, representing possible biomarkers of the disease, their outcomes do not provide a clear answer on which cell subset(s) drive depression. Sørensen et al. described twelve populations upregulated in MDD patients, compared to healthy controls: these include total leukocyte, granulocyte, basophil, neutrophil and monocyte absolute counts, but also more specific subsets such as natural killer (NK) cells, B cells, intermediate monocytes, both naïve and memory CD4^+^ Th cells, immature double positive T cells and CD25^+^ and HLA-DR^+^ activated T cells. Additionally, the ratios between CD4^+^/CD8^+^ cells, neutrophils/lymphocytes and Th17/T regulatory (Tregs) cells were also higher in MDD patients than in controls. On the other hand, a downregulation of CD16^+^ NK cells and NK T cells (NKT) was observed in MDD (159). On the opposite, Foley et al., showed that percentages of lymphocytes and both Th1 and Th2 subsets were downregulated in MDD patients, while total CD4^+^ Th cell counts were increased, compared with healthy controls (148). The discrepancies between these two studies might be due to the methodology, the inclusion criteria, and consequently the number of records included in the meta-analysis (104 vs 27). Of note, not all original research articles converge in the same conclusion: the results hereby reported refer to the pooled standardized mean difference found between MDD patients and healthy controls, in which for the same specific subset each article may report divergent data, which are then grouped and given a different weight, based on sample size. Also, regarding the chosen methodology, while Sørensen et al. used both random- and fixed-effect models, Foley et al. opted only for the fixed-effect meta-analysis, which can contribute to their inconclusive results (148, 159).

**Figure 2:**
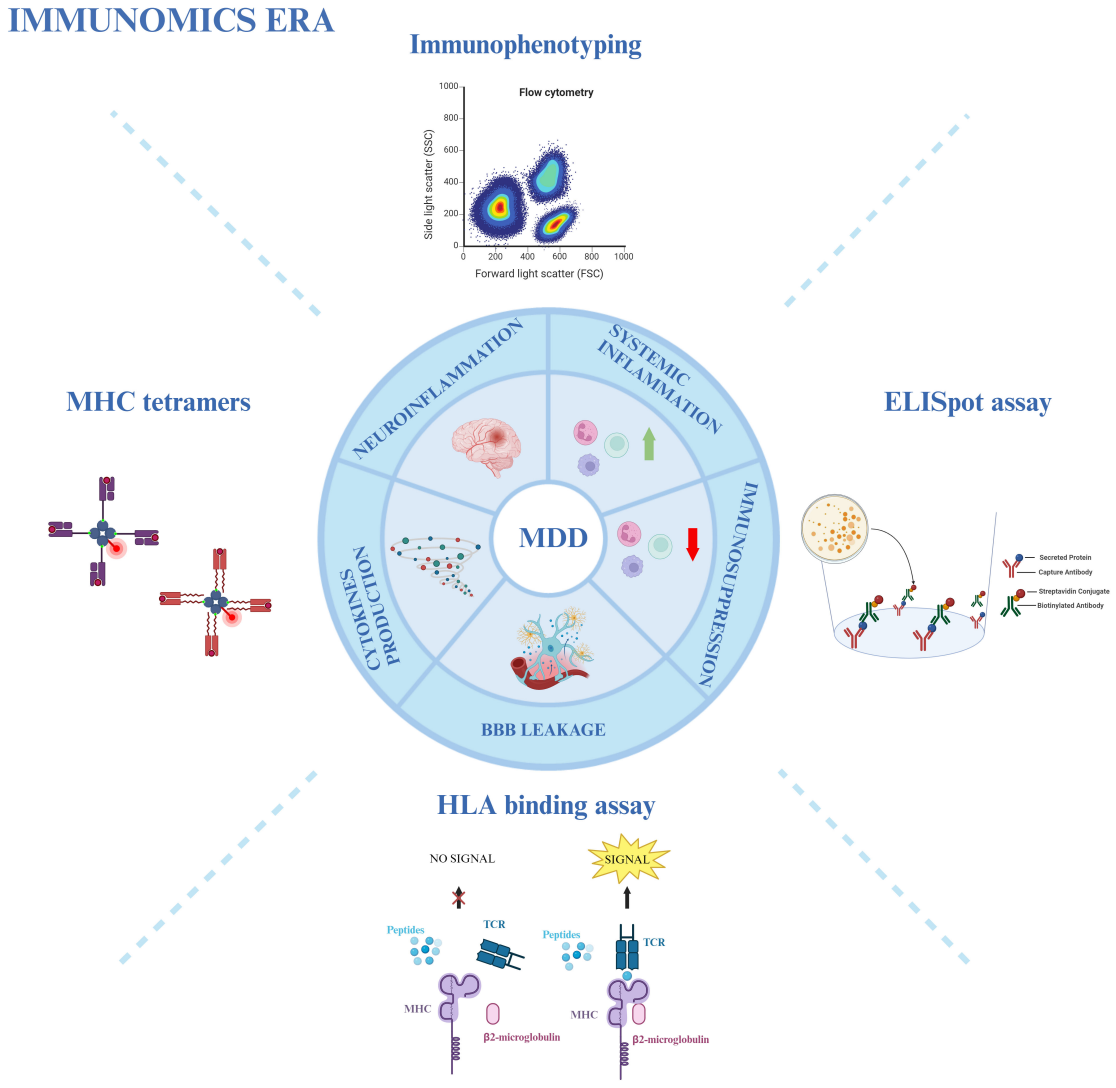
Immunomics approach in MDD. Various immunological techniques provide an overview of the involvement of the immune system in MDD. Immunophenotyping through flow cytometry, MHC tetramers, HLA-binding assays, and ELISpot assays are used to study neuro- and systemic inflammation, immunosuppression, blood-brain barrier (BBB) leakage, and cytokine production in MDD patients. Created with BioRender.com.

Due to the heterogeneity of MDD, a general immune profiling of the disease might not be relevant for treatment choice. As previously introduced, not all MDD patients exhibit a strong immune dysregulation. Interestingly, the culprit subsets can vary even among subjects that demonstrate an exacerbated inflammatory profile. Initially, Lynall et al. showed that a combined analysis of the absolute counts of CD4^+^ Th cells, neutrophils, and eosinophils can accurately distinguish MDD patients from controls; subsequently, within the MDD group, a simultaneous assessment of neutrophils with NKT, and B cells seemed to be the most reliable predictor of disease severity (160). Attempting to categorize the data in an unbiased manner, the authors applied a Gaussian finite multivariate mixture model with a consensus clustering algorithm: this approach revealed four distinct subgroups of patients according to their differential frequency of immune cell populations. While one of the subgroups represented the already described “uninflamed MDD” (58 out of 206 patients), exhibiting low immune cell counts and pro-inflammatory cytokines expression (CRP and IL-6), the other three subgroups represent different inflammatory phenotypes. The rarest profile (10 patients) was enriched in non-classical and intermediate monocytes, eosinophils, B cells, and CD16^high^ NK cells; in contrast, the most common phenotype (100 patients) showed increased counts of lymphoid cells, particularly CD4^+^ and CD8^+^ T lymphocytes and B cells; lastly, the third subgroup (38 patients) displays higher counts of granulocytes and myeloid cells, with the exception of non-classical monocytes (160). Although the authors could not find a correlation between the subgroups and clinical/demographic features, it is clear how important the immune system is in MDD. Therefore, an integrative immunological approach could provide a crucial immunomics overview. This approach utilizes immunophenotyping through flow cytometry, combined with other immunological techniques such as MHC tetramers, ELISpot assay, and HLA-binding assay, to aid in the diagnosis, stratification, and treatment choice for MDD (**Figure 2**) (72, 73, 161).

### 3.4 Microbiota and gut-brain axis - metagenomics in MDD

The connection between the gastrointestinal tract, including gut-associated lymphoid tissue (GALT), the activation of a low-grade inflammatory response, an increased intestinal permeability, and psychiatric symptoms has become more and more clear in recent years (162). The gastrointestinal tract is the interaction site of the immune system with an extensive number of intestinal microbiota, so it may be seen as the “immune gate” of many pathological conditions, including mental disorders like MDD (163).

Gut and brain are able to cross-regulate each other: gut affects brain function, particularly in regions related to stress regulation. In turn, the brain controls motor, sensory, and secretory modalities of the gastrointestinal tract (164). This mutual cross-talk is the so-called gut-brain axis (GBA). The permeability of the gut may be compromised by the diet, antibiotic treatment, stress, or infections (165), allowing antigenic material, such as food-derived antigens or lipopolysaccharides (LPS), to cross to the periphery. This can trigger immune-inflammatory responses, including neuroinflammation through TLRs, which increase the production of pro-inflammatory cytokines like IL-6, IFN-γ, CRP, and TNF-α (166). For example, LPS has been linked to cytokine-mediated illness behavior (described as the coordinated set of behavioral alterations as a result of an infection, caused by pro-inflammatory cytokines (167) and partly superimposable with MDE symptoms), microglial activation, neuronal cell death, and cognitive decline (168). A theory has been proposed defending that exposure to stress through stress hormones, inflammation and autonomic changes (169) modifies brain function by altering the gut microbiome, which in turn alters NLR Family Pyrin Domain Containing 3 (NLRP3) and IL-1β-driven pathways (170). In this vision, microbiome modifications can happen early in the disease and may even be a factor in the initiation of MDD, creating a pathological vicious cycle in which pathogenic changes in MDD over time further contribute to dysbiosis (171).

The interconnection between microbiota and brain can affect MDD patients primarily through the HPA axis, tryptophan metabolites, and microbial products, such as short-chain fatty acids (SCFAs) (like acetate, butyrate, and propionate), indoles (tryptophan metabolites) or through neurotransmitters. Microbiota disbalance increases HPA axis activity in response to stress (172, 173). It can influence serotoninergic, dopaminergic, glutaminergic, noradrenergic, and GABA neurotransmission or, in some cases, bacteria can produce these neurotransmitters by themselves (174). In particular, *Streptococcus*, *Escherichia*, and *Enterococcus* produce serotonin; *Escherichia*, *Bacillus* and *Saccharomyces* produce norepinephrine/dopamine; *Lactobacillus* and *Bifidobacterium* secrete GABA (175). Even if it is unlikely that neurotransmitters produced by microbiota can reach the brain, with the exception of GABA, they can indirectly influence the brain activity through the enteric nervous system (176). Also, tryptophan metabolism pathway can be controlled by products generated by some bacteria, for example *Bifidobacterium infantis* (177, 178). Specifically, SCFAs are not only involved in the BBB integrity, but they can also increase the tryptophan conversion rate into serotonin, indirectly influencing its amount in the brain (179); indeed, SCFAs resulted in being depleted in patients with MDD (180). Moreover, products of tryptophan called indole have been described as neuroactive signaling molecules able to regulate emotional behavior. Indeed, increased tryptophan catabolism into indoles is associated with reduced serotonin availability and increased neuroinflammation. Moreover, several pathways may include the direct effects of indole on central receptors or the activation of the vagus nerve through the influence of gut bacteria stimulating the neuroinflammatory state (173).

In addition, the gut microbiota can modulate distinct subsets of CD4^+^ Th cells through the stimulation of immunological signaling pathways. For example, different studies showed that Tregs and CD4^+^ Th effector cells were decreased in animals receiving long-acting antibiotic treatment. Foxp3^+^ Tregs are defective in germ-free mice, and SFCAs can induce their proliferation instead. Furthermore, *Bacteroides fragilis* stimulate Th1 cell growth (180). Also, segmented filamentous bacteria have been shown to enhance depression susceptibility through the induction of Th17 cells releasing IL-17A (181, 182).

Given all of that, it is becoming evident that it is fundamental to learn more about the landscapes of altered bacteria, bacteriophages, and fecal metabolites as well as their reciprocal interaction in the gut ecosystem of MDD patients, in order to better understand the pathophysiology/pathogenesis and to identify diagnostic and prognostic markers for clinical applications. Combination of metagenomic and metabolomic analyses is the ultimate strategy to explore the taxonomic and functional features of the microbiome (183).

Several studies have shown that patients with depression display abundance in some bacterial species and deficiency in others, compared to healthy individuals (184, 185). Some of them have focused on identifying differential bacteria with a phylogenetic resolution to the genus or family level. Results showed that the majority of the upregulated species in MDD belonged to the phylum Bacteroidetes and Proteobacteria, whereas the major downregulated species belonged to the phylum Firmicutes (186, 187). *Bacteroides* species are particularly important in the interactions with the immune system since they can induce the production of cytokines. On the other hand, beneficial anti-inflammatory effects can be mediated by *Blautia* species, decreased in MDD patients (188).

Furthermore, Zheng and colleagues discovered a unique microbe-based panel that was capable of differentiating between unipolar and bipolar depression, suggesting a specific alteration of the Bacteroidaceae family in patients with MDD as compared to those with BD (189).

In addition to metagenomics, also metabolomics plays an important role in the MDD interaction with the microbiota. The altered metabolites involved in MDD are mainly implicated in amino acid, nucleotide, carbohydrate, and lipid metabolism. In fact, MDD has been linked to significant disruptions in the metabolism of amino acid neurotransmitters, including dopamine, glutamate, and GABA. Moreover, *Bacteroides* and *Blautia* species were found to be altered at genus levels and correlated with amino acid and lipid metabolism (188).

As already mentioned, all these processes lead to depressive- and anxiety-like behavior (170). For this reason, the use of probiotics (living non-pathogenic organisms beneficial for the host), prebiotics (fibers that feed probiotics), and symbiotic (probiotics and prebiotics together) could exhibit beneficial effects in the regulation of depression pathogenesis. A meta-analysis of six trials about the use of probiotics with MDD patients shows a beneficial effect associated with its use in combination with antidepressants (190). In particular, the action of probiotics seems to promote the downregulation of HPA axis, which is overactive in MDD patients (191); in parallel, probiotic administration increases the biosynthesis of GABA (192), as well as upregulates tryptophan production and consequently serotonin availability (178). Some research also indicates that fecal transplantation of microbiota may reduce depressive symptoms and improve quality of life in different clinical samples, including patients with MDD (193–195).

Due to the strength of these techniques, it is highly relevant to link metagenomics and metabolomics with other multi-*omic* techniques, to obtain a complete overview of multifactorial diseases, as is the case of MDD (196).

### 3.5 Neuroimaging biomarkers – image-transcriptome analysis in MDD

Neuroimaging biomarkers are non-invasive intuitive tools that permit the visualization of brain structures and functions *in vivo*, allowing for a better understanding of psychiatric disorders and their treatment response. Moreover, neuroimaging techniques have the advantage of allowing the structural and functional study of the CNS *in vivo*, overcoming the limitation of the post-mortem studies. Various neuroimaging methods used to investigate MDD features include functional MRI (fMRI), magnetic resonance spectroscopy (MRS), PET scan, single photon emission computed tomography (SPECT), EEG, and near-infrared spectroscopy (NIRS) (54, 85). Among these techniques, fMRI is commonly used in both classical neuroimaging and neuroimaging genetics, thus representing an essential tool in personalized medicine in psychiatry (95). By linking genetic variations with protein function, brain structure, structural and functional brain connectivity, and mental manifestations, neuroimaging genetics aims to integrate genetics, psychiatry, and neuroscience. This is achieved by using neuroimaging techniques to quantify biological properties (85, 95, 197). Interestingly, among the neuroimaging techniques, MRS can also be a very reliable and robust metabolomic approach. Under a high magnetic field, nuclei in different chemical groups resonate at slightly different frequencies. This “chemical shift” allows the identification and quantification of the metabolites in different brain areas *in vivo* (198).

Recently, the idea of integrating neuroimaging data with transcriptomic measures has provided unprecedented opportunities for investigating the molecular features that correlate with the organization of brain structures. This image-transcriptome analysis requires three steps: i) processing transcriptional map data, ii) linking expression measurement to neuroimaging phenotypes, and iii) evaluating gene specificity and enrichment (199) and it has been explored to understand structural and functional brain modifications associated with major psychiatric disorders, including MDD (200).

For instance, Sun et al. conducted a combined neuroimaging-transcriptome to explore the genetic mechanisms underlying the cerebral blood flow (CBF) changes in MDD. Their findings highlighted a set of genes expressed in brain tissue, immune cells, and neurons showing correlation with CBF changes in MDD (201). Another example of transcriptome-neuroimaging analysis is provided by a study from Zhu et al, where the authors link macroscopic brain functional changes in MDD patients to specific molecular pathways, which could represent potential targets for antidepressant treatment (202). Furthermore, a recent study by Oh et al. integrates whole exome sequencing (WES) technique and neuroimaging analysis to identify a set of MDD-related genes and recurrent regions of copy number variation, providing insights into the genetic basis of the disease and its neuro-structural alterations (203).

Moreover, recent advancements in neuroimaging and connectome models seem to suggest that multidimensional depression symptoms are linked to alterations of neural network connectivity and organization in different brain areas (67). In this context, functional connectomics based on fMRI could be a promising tool for a more complete understanding of neurobiological mechanisms underlying human brain disorders, and thus a more symptoms-specific personalized treatments.

Overall, these studies integrate analysis from micro to macro level, using multi-scale and multi-*omics* approaches, and combining genetic and neuroimaging information in order to deeply explore the causal relationship between genes, phenotypes, and the environment. This innovative approach represents a promising strategy to elucidate the basis of the pathogenesis of complex diseases, develop targeted therapies, and ultimately improve disease prediction, diagnosis and precision treatment.

## 4 From current standards to system biomedicine

Currently, the established characterization of diseases by clinicians is based on a correlation between pathological analysis and clinical syndromes. The main limitation of this method is that it heavily relies on the available clinical tools and the clinician’s observational skills. This methodology brings several issues such as lack of sensitivity and specificity in identifying and defining disease (204). In response to these shortcomings, a new branch of thought, namely *system biology*, aims at getting rid of the simplistic line of thought, by which a phenotype is a direct consequence of the proteins transcribed by the organisms’ genome, replacing it with the concept of a dynamic network between all the different compartments that compose it (205). The idea that system biology lives upon is that biological systems such as living beings, or even cells themselves, are complex entities that, as such, contract complex diseases (i.e. multifactorial and degenerative ones) which, in order to get treated properly, require complex therapies. This approach may possibly obtain better results than the current method, which focuses on single targets overlooking the remaining components of the network introducing the concept of system biomedicine (SB) (206).

SB aims at translating the data and knowledge derived from the application of system biology into clinical application, with the ultimate goal of improving patients’ quality of life by attributing the most suitable treatment based on their biological background (207). To achieve this goal, there is a need to produce what we could define as a *patient fingerprint*. It is the result of the integration and analysis of very large datasets coming from different advanced technologies capable of representing all of the underlying biological mechanisms of the patients (208). It is directly clear that to obtain this kind of opportunity, there are some fundamental needs that must be taken into consideration: the availability of the data and their dimensionality, methods capable of being adapted to a wide spectrum of applications taking advantage of different quantitative approaches and, in the end, highly characterized systems under physiological conditions so that they can be compared under different circumstances (209). For a better understanding of the complex hierarchy within the different systems, multi-*omic* approaches are required to produce adequate data (210).

**Figure 3:**
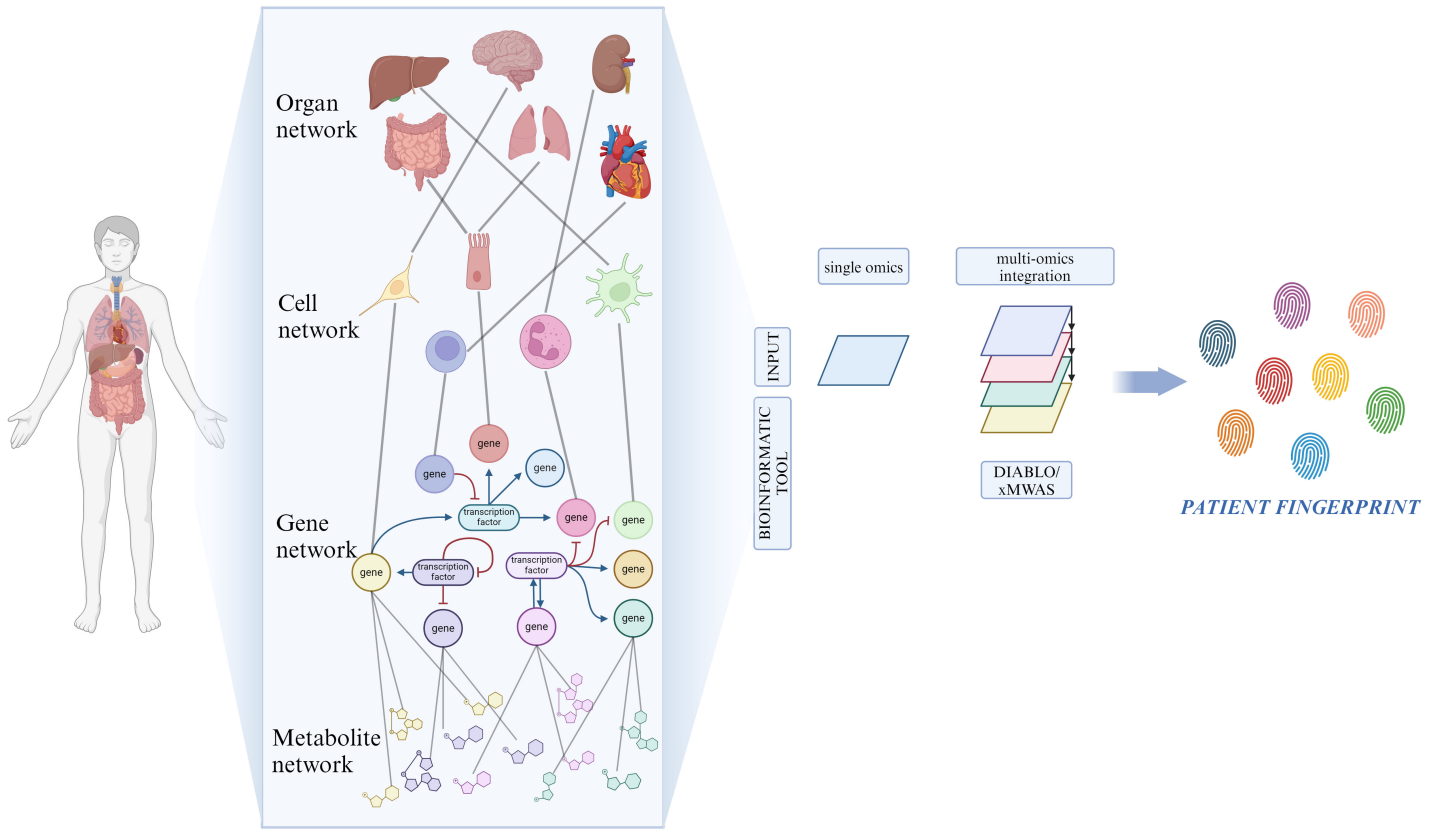
Network for resolving heterogeneity in MDD. On a biological level, our bodies consist of numerous interconnected networks that communicate across various scales (organ, cell, gene and metabolite). Artificial intelligence and machine learning-based bioinformatic tools analyze the role of each single network component through the integration of high-throughput biological data, originated from multi-*omic* techniques. Created with BioRender.com.

A common problem caused by the current level of our technologies comes with the analysis, integration, and understanding of these datasets derived from different *omics.* Considerable amounts of data are being produced compared to our capacity to process them due to the high pace of testing and development of new technologies (211). Many groups have developed tools that attempt to reduce these gaps to answer the need for instruments capable of analyzing and sorting the data. For example, Singh et al. introduced DIABLO, which is a method that relies on the Sparse Generalized Canonical Correlation Analysis technique, capable of selecting correlated variables between different datasets, and on a Projection to Latent Structures technique for effective visualization of the results (212). Another example is the xMWAS tool, which can integrate data from biochemical and phenotypic assays and those deriving from different omics platforms, demonstrating its potential to aid the understanding of the interaction between different systems (**Figure 3**) (213).

### 4.1 System Biomedicine in MDD

Due to the high biological inter-variability within patients of the same groups, concepts such as TRD and Multiple-TRD have been introduced referring to those cases, which account for almost 30% of MDD patients, that fail to achieve clinically significant improvements after different courses of antidepressants (214–216). Through an SB approach, the pathophysiological processes underlying MDD need to be deeply characterized by exploiting high throughput multi-*omic* techniques capable of obtaining complete data from different compartments for good quality analysis to be related with an in-depth clinical characterization of the disorder (217).

To address this problem, various AI-based techniques, such as deep learning algorithms, are being studied for the analysis of very large datasets with the goal of improving patients’ stratification based on their underlying biological features (218). AI is a comprehensive term that includes many different learning models such as Machine Learning (ML), Deep Learning (DL), Natural Language Processing (NLP), and Large Language models (LLM), and all can be adapted to the users need (219). After proper training, their capacity for the analysis of large datasets and pattern recognition can be exploited for them to be used for tasks that range from day-to-day medical diagnostics to much more complex analysis like modern-day *omics* (220). In particular, DL showed a good performance in analyzing multi-*omics* data without the need to reduce the dimensionality of the dataset. This method builds models based on raw data to make predictions or decisions without being explicitly programmed. DL can be unsupervised, for example, for pathway analysis, or supervised, requiring labels for model training to perform classification or regression tasks like patient stratification and treatment response prediction. Supervised DL methods include multilayer perceptron (MLP), convolutional neural network (CNN), and recurrent neural network (RNN) and represent promising tools for multi-*omics* integration in system and precision biomedicine (221).

The main application for the integration of multi-*omic* datasets were performed in oncology mainly in breast and lung cancer to classify tumor subtypes, prognosis, and treatment outcome (221). However, AI introduction in psychiatry may bring a large set of benefits in the choice of the correct therapy and in the guidance of the operator towards the correct diagnosis, which has always been a difficult task, given the overlapping symptoms between different diseases (222–225). This has already been documented showing interesting results in many instances. In the case of schizophrenia, DL, especially in combination with principles from Bayesian statistics, showed good performance in classification and prediction tasks in many neuroimaging studies (226).

In the particular case of MDD, Kleinerman et al. tested the application of latent profile analysis for depression, which allows the subgrouping and clustering of patients’ data coming from different neuroimaging databases according to their underlying condition, to try and predict the outcome of their treatments and to pinpoint the most effective course out of all the available ones. With this strategy, the authors achieved a remission rate of 35.5% over random treatment allocation, achieving better results when compared to the existing state-of-the-art method (i.e. CFRnet, Vulcan, case-based recommender (CBR)) (227).

An example for the integration of heterogeneous data derived from different *omic* and classical approaches could be seen in the work by Li and colleagues (228). The authors analyzed and integrated the data deriving from fecal metagenomic, serum metabolomic, and neuroimaging techniques, from both healthy controls and unmedicated patients with bipolar depression, obtaining a detailed overview of the network concerning the GBA. The results have been analyzed through a random forest technique highlighting the presence of bipolar depression-typical features of brain functionality influenced by neuroactive microbes and metabolites (228). Overall, it is becoming evident how the routes of system biology and SB are promising for the characterization and treatment of these diseases for which traditional methods do not function efficiently.

## 5 Future directions

MDD is a highly prevalent condition often misdiagnosed and with therapy courses that show suboptimal results. As such, the interest in finding a reliable system to address these problems has been constantly increasing, in order to deliver an objective method capable of identifying and treating the disorder (229). The lack of precise algorithms helping clinicians in choosing the appropriate therapy for each patient, leads at the moment to a “trial and error” approach that represents a heavy individual and socio-economic burden (230). Although AI is a technology that is not yet completely reliable for these purposes, some glimpses of it can be seen with the introduction of Woebot, which is an AI-powered automated conversational agent, designed to deliver a cognitive-behavioral therapy comparable to those delivered by therapists. A study regarding the use of Woebot proved that the software has been able to diminish the depressive symptomatology registering, on the other hand, a dropout rate of 17% (222). Other current emerging technologies aim at remotely monitoring different parameters like circadian rhythm, heart rate variations, change in blood pressure, skin temperature and electrodermal activity with the objective of tracking patient’s symptoms through wearable devices, minimizing invasivity while also predicting relapses and treatment response (231–234).

One of the main concerns that come with the introduction and application of AI in medicine is, given the increasing amount of big data, the necessity for robust data protection legislations for individual privacy, along with one for the regulation of its application, which becomes critical when data from AI applications directly impact clinical decision-making. To safeguard principles of medical ethics in the context of AI technology implementation, both the European Union and the United States have already introduced sets of laws: in particular, the General Data Protection Regulation (GDPR) along with the Artificial Intelligence Act (AIA) have been put in effect in the former, while for the latter, the Health Insurance Portability and Accountability Act (HIPAA) has already been established (235–237). Thus, addressing these concerns and improving ethical awareness are first steps towards a responsible and transparent implementation of AI into healthcare practice (219).

## 6 Conclusions

The multifactorial etiology of MDD, involving genetic, environmental, and biological factors, renders its diagnosis, prognosis and treatment quite challenging. We believe that the identification of specific biomarkers for MDD that allow for the selection of the best antidepressant drug for each individual patient and prediction of therapy response represents the most promising approach in order to obtain an objective diagnosis and accurate treatment. Although the use of the currently proposed biomarkers in general clinical practice shows various limitations, the rapid advancements in both *omics* and neuroimaging methodologies, but most of all their possible integration with AI, could lead to a more defined characterization of MDD and to the development of an individualized psychiatric care model. For that, in this review, we have highlighted the potential of applying multi-*omics* as standard clinical practice to develop a more effective diagnostic and therapeutic strategy able to reduce the burden associated with MDD and improve patients’ quality of life, satisfaction, and well-being.

## Author contributions

FS: Writing – original draft, Writing – review & editing, Visualization. HA: Visualization, Writing – original draft, Writing – review & editing. RS: Writing – original draft, Writing – review & editing. SN: Writing – original draft, Writing – review & editing, Visualization. SC: Visualization, Writing – original draft, Writing – review & editing. VL: Writing – original draft, Writing – review & editing. CB: Writing – original draft, Writing – review & editing. GC: Writing – original draft, Writing – review & editing. PR: Writing – original draft, Writing – review & editing, Conceptualization. AC: Funding acquisition, Writing – original draft, Writing – review & editing, Conceptualization.

## Glossary

**Table:**

AI	Artificial Intelligence
AIA	Artificial Intelligence Act
ATAC-seq	Assay for Transposase-Accessible Chromatin using sequencing
BBB	Blood-Brain Barrier
BD	Bipolar Disorder
BDNF	Brain-Derived Neurotrophic Factor
CBF	Cerebral Blood Flow
CBR	Case-Based Recommender
CNN	Convolutional Neural Network
CNS	Central Nervous System
CRP	C-reactive protein
CYP	Cytochrome P450
CyTOF	Cytometry by Time of Flight
DAMPs	Danger/Damage-Associated Molecular Patterns
DL	Deep Learning
DSM	Diagnostic and Statistical Manual
EEG	Electroencephalography
ELISA	Enzyme-linked immunosorbent assay
FACS	Fluorescence-Activated Cell Sorting
fMRI	Functional MRI
GABA	Gamma-Aminobutyric Acid
GALT	Gut-Associated Lymphoid Tissue
GBA	Gut-Brain Axis
GDPR	General Data Protection Regulation
GWAS	Genome-Wide Associating Studies
HIPAA	Health Insurance Portability and Accountability Act
HPA	Hypothalamic–pituitary–adrenal
ICD	International Classification of Diseases
IDO	Indolinime Dioxygenase
IL	Interleukin
LC-MS	Liquid Chromatography-Mass Spectrometry
LLM	Large Language models
LPS	Lipopolysaccharides
MDD	Major Depressive Disorder
MDE	Major Depressive Episode
miRNA	microRNA
ML	Machine Learning
MLP	Multilayer Perceptron
mNGS	Metagenomic Next-Generation Sequencing
MRI	Magnetic Resonance Imaging
mRNA	Messenger RNA
MRS	Magnetic Resonance Spectroscopy
MS	Mass Spectrometry
ncRNA	Non-coding RNA
NGS	Next-Generation Sequencing
NIRS	Near-Infrared Spectroscopy
NK	Natural Killer cells
NKT	Natural Killer T cells
NLP	Natural Language Processing
NLRP3	NLR Family Pyrin Domain Containing 3
PAMPs	Pathogen-Associated Molecular Patterns
PET	Positron Emission Tomography
RNA-seq	RNA sequencing
RNN	Recurrent Neural Network
rRNA	Ribosomal RNA
SB	System Biomedicine
SCFA	Short-Chain Fatty Acids
scRNA-seq	Single cell RNA-sequencing
SNP	Single Nucleotide Polymorphism
snRNA-seq	Single-nucleus RNA-sequencing
SPECT	Single Photon Emission Computed Tomography
Th	T helper
TLR	Toll-Like Receptor
TNF-α	Tumor Necrosis Factor-α
TRD	Treatment-Resistant Depression
Treg	T regulatory cells
WES	Whole Exome Sequencing
WGCNA	Weighted Gene Co-expression Network Analysis
WHO	World Health Organization

## References

[1] Otte C Gold SM Penninx BW Pariante CM Etkin A Fava M. Major depressive disorder. Nat Rev Dis Prim. (2016) 2:1–21. doi: 10.1038/nrdp.2016.65 27629598

[2] Lee MT Peng WH Kan HW Wu CC Wang DW Ho YC. Neurobiology of depression: chronic stress alters the glutamatergic system in the brain—Focusing on AMPA receptor. Biomedicines. (2022) 10:1005. doi: 10.3390/biomedicines10051005 35625742 PMC9138646

[3] Nobis A Zalewski D Waszkiewicz N. Peripheral markers of depression. J Clin Med. (2020) 9:3793. doi: 10.3390/jcm9123793 33255237 PMC7760788

[4] World Health Organization. Global burden of mental disorders and the need for a comprehensive, coordinated response from health and social sectors at the country level - EB130/9. Geneva, Switzerland: Executive Board 130th Session (2011).

[5] Global Burden of Disease Collaborative Network. Global Burden of Disease Study 2019 (GBD 2019) Results. Seattle, United States: Institute for Health Metrics and Evaluation (IHME (2019).

[6] Ferrari AJ Santomauro DF Herrera AMM Shadid J Ashbaugh C Erskine HE. Global, regional, and national burden of 12 mental disorders in 204 countries and territories, 1990–2019: a systematic analysis for the Global Burden of Disease Study 2019. Lancet Psychiatry. (2022) 9:137–50. doi: 10.1016/S2215-0366(21)00395-3 PMC8776563 35026139

[7] Greenberg P Chitnis A Louie D Suthoff E Chen SY Maitland J. The economic burden of adults with major depressive disorder in the United States (2019). Adv Ther. (2023) 40:4460–79. doi: 10.1007/s12325-023-02622-x PMC10499687 37518849

[8] Cai H Xie XM Zhang Q Cui X Lin JX Sim K. Prevalence of suicidality in major depressive disorder: A systematic review and meta-analysis of comparative studies. Front Psychiatry. (2021) 12. doi: 10.3389/fpsyt.2021.690130 PMC8481605 34603096

[9] Nemeroff CB. The state of our understanding of the pathophysiology and optimal treatment of depression: Glass half full or half empty? Am J Psychiatry. (2020) 177:671–85. doi: 10.1176/appi.ajp.2020.20060845 32741287

[10] Pan YJ Yeh LL Chan HY Chang CK. Excess mortality and shortened life expectancy in people with major mental illnesses in Taiwan. Epidemiol Psychiatr Sci. (2020) 29:e156. doi: 10.1017/S2045796020000694 32792024 PMC7443795

[11] König H König HH Konnopka A. The excess costs of depression: A systematic review and meta-analysis. Epidemiol Psychiatr Sci. (2019) 29:e30. doi: 10.1017/S2045796019000180 PMC8061284 30947759

[12] Benacek J Lawal N Ong T Tomasik J Martin-Key NA Funnell EL. Identification of predictors of mood disorder misdiagnosis and subsequent help-seeking behavior in individuals with depressive symptoms: gradient-boosted tree machine learning approach. JMIR Ment Heal. (2024) 11:e50738. doi: 10.2196/50738 PMC10811571 38206660

[13] Maes M Zhou B Jirakran K Vasupanrajit A Boonchaya-Anant P Tunvirachaisakul C. Towards a major methodological shift in depression research by assessing continuous scores of recurrence of illness, lifetime and current suicidal behaviors and phenome features. J Affect Disord. (2024) 350:728–40. doi: 10.1016/j.jad.2024.01.150 38246281

[14] American Psychiatric Association. Diagnostic and Statistical Manual of Mental Disorders: DSM-5™. 5th ed. Washington, DC, US: American Psychiatric Publishing (2013). doi: 10.1176/appi.books.9780890425596

[15] Singh T Rajput M. Misdiagnosis of bipolar disorder. Am J Manag Care. (2006) 10:57–63. PMC2945875 20877548

[16] Maj M Stein DJ Parker G Zimmerman M Fava GA De Hert M. The clinical characterization of the adult patient with depression aimed at personalization of management. World Psychiatry. (2020) 19:269–93. doi: 10.1002/wps.20771 PMC7491646 32931110

[17] Hamilton M. A rating scale for depression. J Neurol Neurosurg Psychiatry. (1960) 23:56–62. doi: 10.1037/t04100-000 PMC495331 14399272

[18] Montgomery SA Asberg M. A new depression scale designed to be sensitive to change. Br J Psychiatry. (1979) 134:382–9. doi: 10.1192/bjp.134.4.382 444788

[19] Beck AT Ward CH Mendelson M Mock J Erbaugh J. An inventory for measuring depression. Arch Gen Psychiatry. (1961) 4:561–71. doi: 10.1001/archpsyc.1961.01710120031004 13688369

[20] Franklin JC Ribeiro JD Fox KR Bentley KH Kleiman EM Huang X. Risk factors for suicidal thoughts and behaviors: A meta-analysis of 50 years of research. Psychol Bull. (2017) 143:187–232. doi: 10.1037/bul0000084 27841450

[21] Posner K Brown GK Stanley B Brent DA Yershova KV Oquendo MA. The Columbia-suicide severity rating scale: Initial validity and internal consistency findings from three multisite studies with adolescents and adults. Am J Psychiatry. (2011) 168:1266–77. doi: 10.1176/appi.ajp.2011.10111704 PMC3893686 22193671

[22] Conradi HJ Ormel J De Jonge P. Presence of individual (residual) symptoms during depressive episodes and periods of remission: A 3-year prospective study. Psychol Med. (2011) 41:1165–74. doi: 10.1017/S0033291710001911 20932356

[23] Morozova A Zorkina Y Abramova O Pavlova O Pavlov K Solovevа K. Neurobiological highlights of cognitive impairment in psychiatric disorders. Int J Mol Sci. (2022) 23:1217. doi: 10.3390/ijms23031217 35163141 PMC8835608

[24] Wen M Dong Z Zhang L Li B Zhang Y Li K. Depression and cognitive impairment: current understanding of its neurobiology and diagnosis. Neuropsychiatr Dis Treat. (2022) 18:2783–94. doi: 10.2147/NDT.S383093 PMC9719265 36471744

[25] Martin DM Wollny-Huttarsch D Nikolin S McClintock SM Alonzo A Lisanby SH. Neurocognitive subgroups in major depressive disorder. Neuropsychology. (2020) 34:726–34. doi: 10.1037/neu0000626 32324004

[26] Cosci F Fava GA. Staging of mental disorders: Systematic review. Psychother Psychosom. (2013) 82:20–34. doi: 10.1159/000342243 23147126

[27] Guidi J Tomba E Cosci F Park SK Fava GA. The role of staging in planning psychotherapeutic interventions in depression. J Clin Psychiatry. (2017) 78:456–63. doi: 10.4088/JCP.16r10736 28297594

[28] Trevino K McClintock SM Fischer NMD Vora A Husain MM. Defining treatment-resistant depression: A comprehensive review of the literature. Ann Clin Psychiatry. (2014) 26:222–32. 25166485

[29] World Health Organization. International statistical classification of diseases and related health problems - 11th revision. World Heal Organ (2021). 10.2471/BLT.25.294650 PMC13531092 42683092

[30] Zimmerman M McGlinchey JB Posternak MA Friedman M Attiullah N Boerescu D. How should remission from depression be defined? The depressed patient’s perspective. Am J Psychiatry. (2006) 163:148–50. doi: 10.1176/appi.ajp.163.1.148 16390903

[31] Demyttenaere K Donneau AF Albert A Ansseau M Constant E Van Heeringen K. What is important in being cured from depression? Discordance between physicians and patients (1). J Affect Disord. (2015) 174:390–6. doi: 10.1016/j.jad.2014.12.004 25545606

[32] IsHak WW Bonifay W Collison K Reid M Youssef H Parisi T. The recovery index: A novel approach to measuring recovery and predicting remission in major depressive disorder. J Affect Disord. (2017) 208:369–74. doi: 10.1016/j.jad.2016.08.081 27810720

[33] Kendall KM Van Assche E Andlauer TFM Choi KW Luykx JJ Schulte EC. The genetic basis of major depression. Psychol Med. (2021) 51:2217–30. doi: 10.1017/S0033291721000441 33682643

[34] Slavich GM Sacher J. Stress, sex hormones, inflammation, and major depressive disorder: Extending Social Signal Transduction Theory of Depression to account for sex differences in mood disorders. Psychopharmacol (Berl). (2019) 236:3063–79. doi: 10.1007/s00213-019-05326-9 PMC6821593 31359117

[35] Lovik A González-Hijón J Hoffart A Fawns-Ritchie C Magnúsdóttir I Lu L. Elevated symptoms of depression and anxiety among family members and friends of critically ill COVID-19 patients – an observational study of five cohorts across four countries. Lancet Reg Heal - Eur. (2023) 33:100733. doi: 10.1016/j.lanepe.2023.100733 PMC10636287 37953992

[36] Cui L Li S Wang S Wu X Liu Y Yu W. Major depressive disorder: hypothesis, mechanism, prevention and treatment. Signal Transduct Target Ther. (2024) 9:30. doi: 10.1038/s41392-024-01738-y 38331979 PMC10853571

[37] Stetler C Miller GE. Depression and hypothalamic-pituitary-adrenal activation: A quantitative summary of four decades of research. Psychosom Med. (2011) 73:114–26. doi: 10.1097/PSY.0b013e31820ad12b 21257974

[38] Milaneschi Y Lamers F Berk M Penninx BWJH. Depression heterogeneity and its biological underpinnings: toward immunometabolic depression. Biol Psychiatry. (2020) 88:369–80. doi: 10.1016/j.biopsych.2020.01.014 32247527

[39] Fries GR Saldana VA Finnstein J Rein T. Molecular pathways of major depressive disorder converge on the synapse. Mol Psychiatry. (2023) 28:284–97. doi: 10.1038/s41380-022-01806-1 PMC9540059 36203007

[40] Voineskos D Daskalakis ZJ Blumberger DM. Management of treatment-resistant depression: Challenges and strategies. Neuropsychiatr Dis Treat. (2020) 16:221–34. doi: 10.2147/NDT PMC6982454 32021216

[41] Zanardi R Prestifilippo D Fabbri C Colombo C Maron E Serretti A. Precision psychiatry in clinical practice. Int J Psychiatry Clin Pract. (2021) 25:19–27. doi: 10.1080/13651501.2020.1809680 32852246

[42] Delpierre C Lefèvre T. Precision and personalized medicine: What their current definition says and silences about the model of health they promote. Implication for the development of personalized health. Front Sociol. (2023) 8. doi: 10.3389/fsoc.2023.1112159 PMC9989160 36895332

[43] Paul GL. Strategy of outcome research in psychotherapy. J Consult Psychol. (1967) 31:109–18. doi: 10.1037/h0024436 5342732

[44] Cohen ZD Derubeis RJ. Treatment selection in depression. Annu Rev Clin Psychol. (2018) 14:209–36. doi: 10.1146/annurev-clinpsy-050817-084746 29494258

[45] Dimitrakopoulos L Prassas I Diamandis EP Charames GS. Onco-proteogenomics: Multi-omics level data integration for accurate phenotype prediction. Crit Rev Clin Lab Sci. (2017) 54:414–32. doi: 10.1080/10408363.2017.1384446 29025326

[46] Grant CW Barreto EF Kumar R Kaddurah-Daouk R Skime M Mayes T. Multi-omics characterization of early-and adult-onset major depressive disorder. J Pers Med. (2022) 12:412. doi: 10.3390/jpm12030412 35330412 PMC8949112

[47] Lake BB Chen S Sos BC Fan J Kaeser GE Yung YC. Integrative single-cell analysis of transcriptional and epigenetic states in the human adult brain. Nat Biotechnol. (2018) 36:70–80. doi: 10.1038/nbt.4038 PMC5951394 29227469

[48] Chen K Baluya D Tosun M Li F Maletic-Savatic M. Imaging mass spectrometry: A new tool to assess molecular underpinnings of neurodegeneration. Metabolites. (2019) 9:135. doi: 10.3390/metabo9070135 31295847 PMC6681116

[49] Sathyanarayanan A Mueller TT Ali Moni M Schueler K Baune BT Lio P. Multi-omics data integration methods and their applications in psychiatric disorders. Eur Neuropsychopharmacol. (2023) 69:26–46. doi: 10.1016/j.euroneuro.2023.01.001 36706689

[50] Detke MJ Wiltse CG Mallinckrodt CH McNamara RK Demitrack MA Bitter I. Duloxetine in the acute and long-term treatment of major depressive disorder: A placebo- and paroxetine-controlled trial. Eur Neuropsychopharmacol. (2004) 14:457–70. doi: 10.1016/j.euroneuro.2004.01.002 15589385

[51] Schork NJ. Personalized medicine: Time for one-person trials. Nature. (2015) 520:609–11. doi: 10.1038/520609a 25925459

[52] Bauer M Rush AJ Ricken R Pilhatsch M Adli M. Algorithms for treatment of major depressive disorder: efficacy and cost-effectiveness. Pharmacopsychiatry. (2019) 52:117–25. doi: 10.1055/a-0643-4830 29986372

[53] Penn E Tracy DK. The drugs don’t work? antidepressants and the current and future pharmacological management of depression. Ther Adv Psychopharmacol. (2012) 2:179–88. doi: 10.1177/2045125312445469 PMC3736946 23983973

[54] Kang SG Cho SE. Neuroimaging biomarkers for predicting treatment response and recurrence of major depressive disorder. Int J Mol Sci. (2020) 21:2148. doi: 10.3390/ijms21062148 32245086 PMC7139562

[55] Howard DM Adams MJ Clarke TK Hafferty JD Gibson J Shirali M. Genome-wide meta-analysis of depression identifies 102 independent variants and highlights the importance of the prefrontal brain regions. Nat Neurosci. (2019) 22:343–52. doi: 10.1038/s41593-018-0326-7 PMC6522363 30718901

[56] Maitra M Mitsuhashi H Rahimian R Chawla A Yang J Fiori LM. Cell type specific transcriptomic differences in depression show similar patterns between males and females but implicate distinct cell types and genes. Nat Commun. (2023) 14:2912. doi: 10.1038/s41467-023-38530-5 37217515 PMC10203145

[57] Zhang L Liu C Li Y Wu Y Wei Y Zeng D. Plasma biomarker panel for major depressive disorder by quantitative proteomics using ensemble learning algorithm: A preliminary study. Psychiatry Res. (2023) 323:115185. doi: 10.1016/j.psychres.2023.115185 37003170

[58] de Kluiver H Jansen R Penninx BWJH Giltay EJ Schoevers RA Milaneschi Y. Metabolomics signatures of depression: the role of symptom profiles. Transl Psychiatry. (2023) 13:198. doi: 10.1038/s41398-023-02484-5 37301859 PMC10257720

[59] Hasin Y Seldin M Lusis A. Multi-omics approaches to disease. Genome Biol. (2017) 18:83. doi: 10.1186/s13059-017-1215-1 28476144 PMC5418815

[60] Bonaguro L Schulte-Schrepping J Ulas T Aschenbrenner AC Beyer M. Schultze JL. A guide to systems-level immunomics. Nat Immunol. (2022) 23:1412–3. doi: 10.1038/s41590-022-01309-9 36138185

[61] Hoffmann A Sportelli V Ziller M Spengler D. Epigenomics of major depressive disorders and schizophrenia: Early life decides. Int J Mol Sci. (2017) 18:1711. doi: 10.3390/ijms18081711 28777307 PMC5578101

[62] Böttcher C Fernández-Zapata C Snijders GJL Schlickeiser S Sneeboer MAM Kunkel D. Single-cell mass cytometry of microglia in major depressive disorder reveals a non-inflammatory phenotype with increased homeostatic marker expression. Transl Psychiatry. (2020) 10:310. doi: 10.1038/s41398-020-00992-2 32917850 PMC7486938

[63] Clish CB. Metabolomics: an emerging but powerful tool for precision medicine. Mol Case Stud. (2015) 1:a000588. doi: 10.1101/mcs.a000588 PMC4850886 27148576

[64] Kumar PS. Microbiomics: Were we all wrong before? Periodontol 2000. (2021) 85:8–11. doi: 10.1111/prd.12373 33226670

[65] Chiu CY Miller SA. Clinical metagenomics. Nat Rev Genet. (2019) 20:341–55. doi: 10.1038/s41576-019-0113-7 PMC6858796 30918369

[66] Thomas T Gilbert J Meyer F. Metagenomics - a guide from sampling to data analysis. Microb Inform Exp. (2012) 2:3. doi: 10.1186/2042-5783-2-3 22587947 PMC3351745

[67] Chai Y Sheline YI Oathes DJ Balderston NL Rao H Yu M. Functional connectomics in depression: insights into therapies. Trends Cognit Sci. (2023) 27:814–32. doi: 10.1016/j.tics.2023.05.006 PMC10476530 37286432

[68] Gong Q He Y. Depression, neuroimaging and connectomics: A selective overview. Biol Psychiatry. (2015) 77:223–35. doi: 10.1016/j.biopsych.2014.08.009 25444171

[69] Siddiqi SH Taylor JJ Horn A Fox MD. Bringing human brain connectomics to clinical practice in psychiatry. Biol Psychiatry. (2023) 93:386–7. doi: 10.1016/j.biopsych.2022.05.026 PMC10184878 35868885

[70] Flores M Glusman G Brogaard K Price ND Hood L. P4 medicine: How systems medicine will transform the healthcare sector and society. Per Med. (2013) 10:565–76. doi: 10.2217/pme.13.57 PMC4204402 25342952

[71] Herrera G Diaz L Martinez-Romero A Gomes A Villamón E Callaghan RC. Cytomics: A multiparametric, dynamic approach to cell research. Toxicol Vitr. (2007) 21:176–82. doi: 10.1016/j.tiv.2006.07.003 16934431

[72] De Sousa KP Doolan DL. Immunomics: A 21st century approach to vaccine development for complex pathogens. Parasitology. (2016) 143:236–44. doi: 10.1017/S0031182015001079 26864136

[73] Tay SH Yaung KN Leong JY Yeo JG Arkachaisri T Albani S. Immunomics in pediatric rheumatic diseases. Front Med. (2019) 6. doi: 10.3389/fmed.2019.00111 PMC6558393 31231652

[74] Beurel E Toups M Nemeroff CB. The bidirectional relationship of depression and inflammation: double trouble. Neuron. (2020) 107:234–56. doi: 10.1016/j.neuron.2020.06.002 PMC7381373 32553197

[75] Gibney SM Drexhage HA. Evidence for a dysregulated immune system in the etiology of psychiatric disorders. J Neuroimmune Pharmacol. (2013) 8:900–20. doi: 10.1007/s11481-013-9462-8 23645137

[76] Müller N. Immunology of major depression. Neuroimmunomodulation. (2014) 21:123–30. doi: 10.1159/000356540 24557045

[77] Scala JJ Ganz AB Snyder MP. Precision medicine approaches to mental health care. Physiology. (2023) 38:82–98. doi: 10.1152/physiol.00013.2022 PMC9870582 36099270

[78] Zhdanava M Pilon D Ghelerter I Chow W Joshi K Lefebvre P. The prevalence and national burden of treatment-resistant depression and major depressive disorder in the United States. J Clin Psychiatry. (2021) 82:29169. doi: 10.4088/JCP.20m13699 33989464

[79] Prendes-Alvarez S Nemeroff CB. Personalized medicine: Prediction of disease vulnerability in mood disorders. Neurosci Lett. (2018) 669:10–3. doi: 10.1016/j.neulet.2016.09.049 27746310

[80] Kapur S Phillips AG Insel TR. Why has it taken so long for biological psychiatry to develop clinical tests and what to do about it. Mol Psychiatry. (2012) 17:1174–9. doi: 10.1038/mp.2012.105 22869033

[81] Strawbridge R Arnone D Danese A Papadopoulos A Herane Vives A Cleare AJ. Inflammation and clinical response to treatment in depression: A meta-analysis. Eur Neuropsychopharmacol. (2015) 25:1532–43. doi: 10.1016/j.euroneuro.2015.06.007 26169573

[82] Atkinson AJ Colburn WA DeGruttola VG DeMets DL Downing GJ Hoth DF. Biomarkers and surrogate endpoints: Preferred definitions and conceptual framework. Clin Pharmacol Ther. (2001) 69:89–95. doi: 10.1067/mcp.2001.113989 11240971

[83] Lopresti AL Maker GL Hood SD Drummond PD. A review of peripheral biomarkers in major depression: The potential of inflammatory and oxidative stress biomarkers. Prog Neuropsychopharmacol Biol Psychiatry. (2014) 48:102–11. doi: 10.1016/j.pnpbp.2013.09.017 24104186

[84] Kennis M Gerritsen L van Dalen M Williams A Cuijpers P Bockting C. Prospective biomarkers of major depressive disorder: a systematic review and meta-analysis. Mol Psychiatry. (2020) 25:321–38. doi: 10.1038/s41380-019-0585-z PMC6974432 31745238

[85] Dadkhah M Jafarzadehgharehziaaddin M Molaei S Akbari M Gholizadeh N Fathi F. Major depressive disorder: Biomarkers and biosensors. Clin Chim Acta. (2023) 547:117437. doi: 10.1016/j.cca.2023.117437 37315724

[86] Osimo EF Pillinger T Rodriguez IM Khandaker GM Pariante CM Howes OD. Inflammatory markers in depression: A meta-analysis of mean differences and variability in 5,166 patients and 5,083 controls. Brain Behav Immun. (2020) 87:901–9. doi: 10.1016/j.bbi.2020.02.010 PMC7327519 32113908

[87] Goldsmith DR Rapaport MH Miller BJ. A meta-analysis of blood cytokine network alterations in psychiatric patients: Comparisons between schizophrenia, bipolar disorder and depression. Mol Psychiatry. (2016) 21:1696–709. doi: 10.1038/mp.2016.3 PMC6056174 26903267

[88] Xu Y Liang J Sun Y Zhang Y Shan F Ge J. Serum cytokines-based biomarkers in the diagnosis and monitoring of therapeutic response in patients with major depressive disorder. Int Immunopharmacol. (2023) 118:110108. doi: 10.1016/j.intimp.2023.110108 37004349

[89] Ait Tayeb AEK Poinsignon V Chappell K Bouligand J Becquemont L Verstuyft C. Major depressive disorder and oxidative stress: A review of peripheral and genetic biomarkers according to clinical characteristics and disease stages. Antioxidants. (2023) 12:942. doi: 10.3390/antiox12040942 37107318 PMC10135827

[90] Silva-Costa LC Smith BJ Carregari VC Souza GHMF Vieira EM Mendes-Silva AP. Plasma proteomic signature of major depressive episode in the elderly. J Proteomics. (2022) 269:104713. doi: 10.1016/j.jprot.2022.104713 36058540

[91] Schubert KO Stacey D Arentz G Clark SR Air T Hoffmann P. Targeted proteomic analysis of cognitive dysfunction in remitted major depressive disorder: Opportunities of multi-omics approaches towards predictive, preventive, and personalized psychiatry. J Proteomics. (2018) 188:63–70. doi: 10.1016/j.jprot.2018.02.023 29474866

[92] MacDonald K Krishnan A Cervenka E Hu G Guadagno E Trakadis Y. Biomarkers for major depressive and bipolar disorders using metabolomics: A systematic review. Am J Med Genet Part B Neuropsychiatr Genet. (2019) 180:122–37. doi: 10.1002/ajmg.b.32680 30411484

[93] Wolfender JL Marti G Thomas A Bertrand S. Current approaches and challenges for the metabolite profiling of complex natural extracts. J Chromatogr A. (2015) 1382:136–64. doi: 10.1016/j.chroma.2014.10.091 25464997

[94] Pan JX Xia JJ Deng FL Liang WW Wu J Yin BM. Diagnosis of major depressive disorder based on changes in multiple plasma neurotransmitters: A targeted metabolomics study. Transl Psychiatry. (2018) 8:130. doi: 10.1038/s41398-018-0183-x 29991685 PMC6039504

[95] Ozomaro U Wahlestedt C Nemeroff CB. Personalized medicine in psychiatry: Problems and promises. BMC Med. (2013) 11:132. doi: 10.1186/1741-7015-11-132 23680237 PMC3668172

[96] Lin E Lin CH Lane HY. Precision psychiatry applications with pharmacogenomics: Artificial intelligence and machine learning approaches. Int J Mol Sci. (2020) 21:969. doi: 10.3390/ijms21030969 32024055 PMC7037937

[97] Miller DB O’Callaghan JP. Personalized medicine in major depressive disorder - Opportunities and pitfalls. Metabolism. (2013) 62:S34–9. doi: 10.1016/j.metabol.2012.08.021 PMC4672728 23021040

[98] Benedetti F Colombo C Pirovano A Marino E Smeraldi E. The catechol-O-methyltransferase Val(108/158)Met polymorphism affects antidepressant response to paroxetine in a naturalistic setting. Psychopharmacol (Berl). (2009) 203:155–60. doi: 10.1007/s00213-008-1381-7 18989660

[99] Tsai SJ Gau YTA Hong CJ Liou YJ Yu YWY Chen TJ. Sexually dimorphic effect of catechol-O-methyltransferase val158met polymorphism on clinical response to fluoxetine in major depressive patients. J Affect Disord. (2009) 113:183–7. doi: 10.1016/j.jad.2008.04.017 18533273

[100] Domschke K Hohoff C Mortensen LS Roehrs T Deckert J Arolt V. Monoamine oxidase A variant influences antidepressant treatment response in female patients with Major Depression. Prog Neuropsychopharmacol Biol Psychiatry. (2008) 32:224–8. doi: 10.1016/j.pnpbp.2007.08.011 17884271

[101] Yu YWY Tsai SJ Hong CJ Chen TJ Chen MC Yang CW. Association study of a Monoamine oxidase A gene promoter polymorphism with major depressive disorder and antidepressant response. Neuropsychopharmacology. (2005) 30:1719–23. doi: 10.1038/sj.npp.1300785 15956990

[102] Tadić A Müller MJ Rujescu D Kohnen H Stassen HH Dahmen N. The MAOA T941G polymorphism and short-term treatment response to mirtazapine and paroxetine in major depression. Am J Med Genet Part B Neuropsychiatr Genet. (2007) 144:325–31. doi: 10.1002/ajmg.b.30462 17192957

[103] Singh AB Bousman CA Ng CH Byron K Berk M. ABCB1 polymorphism predicts escitalopram dose needed for remission in major depression. Transl Psychiatry. (2012) 2:e198. doi: 10.1038/tp.2012.115 23188198 PMC3565756

[104] Uhr M Tontsch A Namendorf C Ripke S Lucae S Ising M. Polymorphisms in the drug transporter gene ABCB1 predict antidepressant treatment response in depression. Neuron. (2008) 57:203–9. doi: 10.1016/j.neuron.2007.11.017 18215618

[105] Kato M Fukuda T Serretti A Wakeno M Okugawa G Ikenaga Y. ABCB1 (MDR1) gene polymorphisms are associated with the clinical response to paroxetine in patients with major depressive disorder. Prog Neuropsychopharmacol Biol Psychiatry. (2008) 32:398–404. doi: 10.1016/j.pnpbp.2007.09.003 17913323

[106] Mayer ML. GRIK4 and the kainate receptor. Am J Psychiatry. (2007) 164:11849–53. doi: 10.1176/appi.ajp.2007.07060996 17671275

[107] Stawski P Janovjak H Trauner D. Pharmacology of ionotropic glutamate receptors: A structural perspective. Bioorganic Med Chem. (2010) 18:7759–72. doi: 10.1016/j.bmc.2010.09.012 20947363

[108] Alqahtani AM Kumarappan C Kumar V Srinivasan R Krishnaraju V. Understanding the genetic aspects of resistance to antidepressants treatment. Eur Rev Med Pharmacol Sci. (2020) 24:7784–95. doi: 10.26355/eurrev_202007_22281 32744705

[109] Hacimusalar Y Eşel E. Suggested biomarkers for major depressive disorder. Noropsikiyatri Ars. (2018) 55:280–90. doi: 10.29399/npa.19482 PMC6138223 30224877

[110] Tubbs JD Ding J Baum L Sham PC. Immune dysregulation in depression: Evidence from genome-wide association. Brain Behav Immun - Heal. (2020) 7:100108. doi: 10.1016/j.bbih.2020.100108 PMC8474691 34589869

[111] Grasmäder K Verwohlt PL Rietschel M Dragicevic A Müller M Hiemke C. Impact of polymorphisms of cytochrome-P450 isoenzymes 2C9, 2C19 and 2D6 on plasma concentrations and clinical effects of antidepressants in a naturalistic clinical setting. Eur J Clin Pharmacol. (2004) 60:329–36. doi: 10.1007/s00228-004-0766-8 15168101

[112] Kumar V Wahlstrom JL Rock DA Warren CJ Gorman LA Tracy TS. CYP2C9 inhibition: Impact of probe selection and pharmacogenetics on in vitro inhibition profiles. Drug Metab Dispos. (2006) 34:1966–75. doi: 10.1124/dmd.106.010926 16963489

[113] Zanger UM Schwab M. Cytochrome P450 enzymes in drug metabolism: Regulation of gene expression, enzyme activities, and impact of genetic variation. Pharmacol Ther. (2013) 138:103–41. doi: 10.1016/j.pharmthera.2012.12.007 23333322

[114] Kirchheiner J Nickchen K Bauer M Wong ML Licinio J Roots I. Pharmacogenetics of antidepressants and antipsychotics: The contribution of allelic variations to the phenotype of drug response. Mol Psychiatry. (2004) 9:442–73. doi: 10.1038/sj.mp.4001494 15037866

[115] Yuan H Mischoulon D Fava M Otto MW. Circulating microRNAs as biomarkers for depression: Many candidates, few finalists. J Affect Disord. (2018) 233:68–78. doi: 10.1016/j.jad.2017.06.058 28673667

[116] Liang Y Zhao G Sun R Mao Y Li G Chen X. Genetic variants in the promoters of let-7 family are associated with an increased risk of major depressive disorder. J Affect Disord. (2015) 183:295–9. doi: 10.1016/j.jad.2015.04.035 26047307

[117] Chuen Lin C Te Lee C Hsiang Sun M Lai Huang T. Increased Levels of miR-30e, miR-132, miR-185, and miR- 212 at Baseline and Increased Brain-derived Neurotrophic Factor Protein and mRNA Levels after Treatment in Patients with Major Depressive Disorder. Neuropsy (London). (2017) 07:920–6. doi: 10.4172/Neuropsychiatry

[118] Amasi-Hartoonian N Pariante CM Cattaneo A Sforzini L. Understanding treatment-resistant depression using “omics” techniques: A systematic review. J Affect Disord. (2022) 318:423–55. doi: 10.1016/j.jad.2022.09.011 36103934

[119] Gururajan A Naughton ME Scott KA O’Connor RM Moloney G Clarke G. MicroRNAs as biomarkers for major depression: A role for let-7b and let-7c. Transl Psychiatry. (2016) 6:e862. doi: 10.1038/tp.2016.131 27483380 PMC5022079

[120] Nagy C Maitra M Tanti A Suderman M Théroux JF Davoli MA. Single-nucleus transcriptomics of the prefrontal cortex in major depressive disorder implicates oligodendrocyte precursor cells and excitatory neurons. Nat Neurosci. (2020) 23:771–81. doi: 10.1038/s41593-020-0621-y 32341540

[121] Wu J Li Y Huang Y Liu L Zhang H Nagy C. Integrating spatial and single-nucleus transcriptomic data elucidates microglial-specific responses in female cynomolgus macaques with depressive-like behaviors. Nat Neurosci. (2023) 26:1352–64. doi: 10.1038/s41593-023-01379-4 37443281

[122] Kohler O Krogh J Mors O Eriksen Benros M. Inflammation in depression and the potential for anti-inflammatory treatment. Curr Neuropharmacol. (2016) 14:732–42. doi: 10.2174/1570159X14666151208113700 PMC5050394 27640518

[123] Dickens C McGowan L Clark-Carter D Creed F. Depression in rheumatoid arthritis: A systematic review of the literature with meta-analysis. Psychosom Med. (2002) 64:52–60. doi: 10.1097/00006842-200201000-00008 11818586

[124] Dowlati Y Herrmann N Swardfager W Liu H Sham L Reim EK. A meta-analysis of cytokines in major depression. Biol Psychiatry. (2010) 67:446–57. doi: 10.1016/j.biopsych.2009.09.033 20015486

[125] Nery FG Monkul ES Hatch JP Fonseca M Zunta-Soares GB Frey BN. Celecoxib as an adjunct in the treatment of depressive or mixed episodes of bipolar disorder: A double-blind, randomized, placebo-controlled study. Hum Psychopharmacol. (2008) 23:87–94. doi: 10.1002/hup.912 18172906

[126] Felger JC Miller AH. Identifying immunophenotypes of inflammation in depression: dismantling the monolith. Biol Psychiatry. (2020) 88:136–8. doi: 10.1016/j.biopsych.2020.04.024 32616200

[127] Leonard B E. The concept of depression as a dysfunction of the immune system. Curr Immunol Rev. (2010) 6:205–12. doi: 10.2174/157339510791823835 PMC3002174 21170282

[128] Kim YK Won E. The influence of stress on neuroinflammation and alterations in brain structure and function in major depressive disorder. Behav Brain Res. (2017) 329:6–11. doi: 10.1016/j.bbr.2017.04.020 28442354

[129] Blume J Douglas SD Evans DL. Immune suppression and immune activation in depression. Brain Behav Immun. (2011) 25:221–9. doi: 10.1016/j.bbi.2010.10.008 PMC3025086 20955778

[130] Zhang JM An J. Cytokines, inflammation, and pain. Int Anesthesiol Clin. (2007) 45:27–37. doi: 10.1097/AIA.0b013e318034194e PMC2785020 17426506

[131] Himmerich H Patsalos O Lichtblau N Ibrahim MAA Dalton B. Cytokine research in depression: Principles, challenges, and open questions. Front Psychiatry. (2019) 10. doi: 10.3389/fpsyt.2019.00030 PMC6374304 30792669

[132] Ruiz NAL Del Ángel DS Brizuela NO Peraza AV Olguín HJ Soto MP. Inflammatory process and immune system in major depressive disorder. Int J Neuropsychopharmacol. (2022) 25:46–53 doi: 10.1093/ijnp/pyab072 PMC8756095 34724041

[133] Deverman BE Patterson PH. Cytokines and CNS development. Neuron. (2009) 64:61–78. doi: 10.1016/j.neuron.2009.09.002 19840550

[134] Stephan AH Barres BA Stevens B. The complement system: An unexpected role in synaptic pruning during development and disease. Annu Rev Neurosci. (2012) 35:369–89. doi: 10.1146/annurev-neuro-061010-113810 22715882

[135] Ruiz NAL Del Ángel DS Olguín HJ Silva ML. Neuroprogression: The hidden mechanism of depression. Neuropsychiatr Dis Treat. (2018) 14:2837–45. doi: 10.2147/NDT PMC6214587 30464468

[136] Pollmächer T Haack M Schuld A Reichenberg A Yirmiya R. Low levels of circulating inflammatory cytokines - Do they affect human brain functions? Brain Behav Immun. (2002) 16:525–32. doi: 10.1016/s0889-1591(02)00004-1 12401466

[137] Capuron L Miller AH. Immune system to brain signaling: Neuropsychopharmacological implications. Pharmacol Ther. (2011) 130:226–38. doi: 10.1016/j.pharmthera.2011.01.014 PMC3072299 21334376

[138] Kiank C Zeden JP Drude S Domanska G Fusch G Otten W. Psychological stress-induced, IDO1-dependent tryptophan catabolism: Implications on immunosuppression in mice and humans. PloS One. (2010) 5:e11825. doi: 10.1371/journal.pone.0011825 20689575 PMC2911374

[139] Savitz J. The kynurenine pathway: a finger in every pie. Mol Psychiatry. (2020) 25:131–47. doi: 10.1038/s41380-019-0414-4 PMC6790159 30980044

[140] Dantzer R. Role of the kynurenine metabolism pathway in inflammation-induced depression: Preclinical approaches. Curr Topics Behav Neurosci. (2017) 31:117–38. doi: 10.1007/978-3-319-51152-8 PMC6585430 27225497

[141] Garrison AM Parrott JM Tuñon A Delgado J Redus L O’Connor JC. Kynurenine pathway metabolic balance influences microglia activity: Targeting kynurenine monooxygenase to dampen neuroinflammation. Psychoneuroendocrinology. (2018) 94:1–10. doi: 10.1016/j.psyneuen.2018.04.019 PMC5995655 29734055

[142] Kindler J Lim CK Weickert CS Boerrigter D Galletly C Liu D. Dysregulation of kynurenine metabolism is related to proinflammatory cytokines, attention, and prefrontal cortex volume in schizophrenia. Mol Psychiatry. (2020) 25:2860–72. doi: 10.1038/s41380-019-0401-9 PMC7577855 30940904

[143] van Velzen LS Wijdeveld M Black CN van Tol MJ van der Wee NJA Veltman DJ. Oxidative stress and brain morphology in individuals with depression, anxiety and healthy controls. Prog Neuropsychopharmacol Biol Psychiatry. (2017) 76:140–4. doi: 10.1016/j.pnpbp.2017.02.017 28249819

[144] Hernández HC Coronel PL Aguilar JC Rodríguez EC. Neurobiology of major depression and its pharmacological treatment. Salud Ment. (2016) 39:47–58. doi: 10.17711/SM.0185-3325.2015.067

[145] Guo H Callaway JB Ting JPY. Inflammasomes: Mechanism of action, role in disease, and therapeutics. Nat Med. (2015) 21:677–87. doi: 10.1038/nm.3893 PMC4519035 26121197

[146] Zhu W Cao FS Feng J Chen HW Wan JR Lu Q. NLRP3 inflammasome activation contributes to long-term behavioral alterations in mice injected with lipopolysaccharide. Neuroscience. (2017) 343:77–84. doi: 10.1016/j.neuroscience.2016.11.037 PMC5349320 27923741

[147] Raison CL Miller AH. Pathogen-host defense in the evolution of depression: insights into epidemiology, genetics, bioregional differences and female preponderance. Neuropsychopharmacology. (2017) 42:5–27. doi: 10.1038/npp.2016.194 PMC5143499 27629366

[148] Foley ÉM Parkinson JT Mitchell RE Turner L Khandaker GM. Peripheral blood cellular immunophenotype in depression: a systematic review and meta-analysis. Mol Psychiatry. (2023) 28:1004–19. doi: 10.1038/s41380-022-01919-7 PMC10005954 36577838

[149] Colonna M Butovsky O. Microglia function in the central nervous system during health and neurodegeneration. Annu Rev Immunol. (2017) 35:441–68. doi: 10.1146/annurev-immunol-051116-052358 PMC8167938 28226226

[150] Kipnis J. Multifaceted interactions between adaptive immunity and the central nervous system. Science. (2016) 353:766–71. doi: 10.1126/science.aag2638 PMC5590839 27540163

[151] Varatharaj A Galea I. The blood-brain barrier in systemic inflammation. Brain Behav Immun. (2017) 60:1–12. doi: 10.1016/j.bbi.2016.03.010 26995317

[152] Hughes V. Microglia: The constant gardeners. Nature. (2012) 485:570–72. doi: 10.1038/485570a 22660301

[153] Schmidt SI Bogetofte H Ritter L Agergaard JB Hammerich D Kabiljagic AA. Microglia-secreted factors enhance dopaminergic differentiation of tissue- and iPSC-derived human neural stem cells. Stem Cell Rep. (2021) 16:281–94. doi: 10.1016/j.stemcr.2020.12.011 PMC7878834 33482100

[154] Sevenich L. Brain-resident microglia and blood-borne macrophages orchestrate central nervous system inflammation in neurodegenerative disorders and brain cancer. Front Immunol. (2018) 9. doi: 10.3389/fimmu.2018.00697 PMC5897444 29681904

[155] Tang Y Le W. Differential roles of M1 and M2 microglia in neurodegenerative diseases. Mol Neurobiol. (2016) 53:1181–94. doi: 10.1007/s12035-014-9070-5 25598354

[156] Singhal G Baune BT. Microglia: An interface between the loss of neuroplasticity and depression. Front Cell Neurosci. (2017) 11:270. doi: 10.3389/fncel.2017.00270 28943841 PMC5596091

[157] Kim YK Na KS. Role of glutamate receptors and glial cells in the pathophysiology of treatment-resistant depression. Prog Neuropsychopharmacol Biol Psychiatry. (2016) 70:117–26. doi: 10.1016/j.pnpbp.2016.03.009 27046518

[158] Kim YK Na KS Myint AM Leonard BE. The role of pro-inflammatory cytokines in neuroinflammation, neurogenesis and the neuroendocrine system in major depression. Prog Neuropsychopharmacol Biol Psychiatry. (2016) 64:277–84. doi: 10.1016/j.pnpbp.2015.06.008 26111720

[159] Sørensen NV Frandsen BH Orlovska-Waast S Buus TB Ødum N Christensen RH. Immune cell composition in unipolar depression: a comprehensive systematic review and meta-analysis. Mol Psychiatry. (2023) 28:391–401. doi: 10.1038/s41380-022-01905-z 36517638

[160] Lynall ME Turner L Bhatti J Cavanagh J de Boer P Mondelli V. Peripheral blood cell–stratified subgroups of inflamed depression. Biol Psychiatry. (2020) 88:185–96. doi: 10.1016/j.biopsych.2019.11.017 32000983

[161] De Groot AS. Immunomics: Discovering new targets for vaccines and therapeutics. Drug Discovery Today. (2006) 11:203–9. doi: 10.1016/S1359-6446(05)03720-7 16580597

[162] Rudzki L Maes M. From “Leaky gut” to impaired glia-neuron communication in depression. Adv Exp Med Biol. (2021) 1305:129–55. doi: 10.1007/978-981-33-6044-0_9 33834399

[163] Rudzki L Szulc A. “Immune Gate” of psychopathology-The role of gut derived immune activation in major psychiatric disorders. Front Psychiatry. (2018) 9. doi: 10.3389/fpsyt.2018.00205 PMC5987016 29896124

[164] Dinan TG Cryan JF. Regulation of the stress response by the gut microbiota: Implications for psychoneuroendocrinology. Psychoneuroendocrinology. (2012) 37:1369–78. doi: 10.1016/j.psyneuen.2012.03.007 22483040

[165] Bercik P Park AJ Sinclair D Khoshdel A Lu J Huang X. The anxiolytic effect of Bifidobacterium longum NCC3001 involves vagal pathways for gut-brain communication. Neurogastroenterol Motil. (2011) 23:1132–9. doi: 10.1111/nmo.2011.23.issue-12 PMC3413724 21988661

[166] Rudzki L Maes M. The microbiota-gut-immune-glia (MGIG) axis in major depression. Mol Neurobiol. (2020) 57:4269–95. doi: 10.20944/preprints202002.0084.v1 32700250

[167] Dantzer R. Cytokine-induced sickness behavior: Where do we stand? Brain Behav Immun. (2001) 15:7–24. doi: 10.1006/brbi.2000.0613 11259077

[168] Zhao J Bi W Xiao S Lan X Cheng X Zhang J. Neuroinflammation induced by lipopolysaccharide causes cognitive impairment in mice. Sci Rep. (2019) 9:5790. doi: 10.1038/s41598-019-42286-8 30962497 PMC6453933

[169] Madison A Kiecolt-Glaser JK. Stress, depression, diet, and the gut microbiota: human–bacteria interactions at the core of psychoneuroimmunology and nutrition. Curr Opin Behav Sci. (2019) 28:105–10. doi: 10.1016/j.cobeha.2019.01.011 PMC7213601 32395568

[170] Inserra A Rogers GB Licinio J Wong ML. The microbiota-inflammasome hypothesis of major depression. BioEssays. (2018) 40:e1800027. doi: 10.1002/bies.201800027 30004130 PMC12180310

[171] Chidambaram SB Essa MM Rathipriya AG Bishir M Ray B Mahalakshmi AM. Gut dysbiosis, defective autophagy and altered immune responses in neurodegenerative diseases: Tales of a vicious cycle. Pharmacol Ther. (2022) 231:107988. doi: 10.1016/j.pharmthera.2021.107988 34536490

[172] Cryan JF O’riordan KJ Cowan CSM Sandhu KV Bastiaanssen TFS Boehme M. The microbiota-gut-brain axis. Physiol Rev. (2019) 99:1877–2013. doi: 10.1152/physrev.00018.2018 31460832

[173] Caspani G Kennedy S Foster JA Swann J. Gut microbial metabolites in depression: Understanding the biochemical mechanisms. Microb Cell. (2019) 6:454–81. doi: 10.15698/mic PMC6780009 31646148

[174] Socała K Doboszewska U Szopa A Serefko A Włodarczyk M Zielińska A. The role of microbiota-gut-brain axis in neuropsychiatric and neurological disorders. Pharmacol Res. (2021) 172:105840. doi: 10.1016/j.phrs.2021.105840 34450312

[175] Holzer P Farzi A. Neuropeptides and the microbiota- Gut-brain axis. Adv Exp Med Biol. (2014) 817:195–219. doi: 10.1007/978-1-4939-0897-4_9 PMC4359909 24997035

[176] De Caro C Iannone LF Citraro R Striano P De Sarro G Constanti A. Can we ‘seize’ the gut microbiota to treat epilepsy? Neurosci Biobehav Rev. (2019) 107:750–64. doi: 10.1016/j.neubiorev.2019.10.002 31626816

[177] Carlessi AS Borba LA Zugno AI Quevedo J Réus GZ. Gut microbiota–brain axis in depression: The role of neuroinflammation. Eur J Neurosci. (2021) 53:222–35. doi: 10.1111/ejn.14631 31785168

[178] Desbonnet L Garrett L Clarke G Bienenstock J Dinan TG. The probiotic Bifidobacteria infantis: An assessment of potential antidepressant properties in the rat. J Psychiatr Res. (2008) 43:164–74. doi: 10.1016/j.jpsychires.2008.03.009 18456279

[179] Reigstad CS Salmonson CE Rainey JF Szurszewski JH Linden DR Sonnenburg JL. Gut microbes promote colonic serotonin production through an effect of short-chain fatty acids on enterochromaffin cells. FASEB J. (2015) 29:1395–403. doi: 10.1096/fj.14-259598 PMC4396604 25550456

[180] Liu L Wang H Chen X Zhang Y Zhang H Xie P. Gut microbiota and its metabolites in depression: from pathogenesis to treatment. eBioMedicine. (2023) 90:104527. doi: 10.1016/j.ebiom.2023.104527 36963238 PMC10051028

[181] Medina-Rodriguez EM Madorma D O’Connor G Mason BL Han D Deo SK. Identification of a signaling mechanism by which the microbiome regulates Th17 cell-mediated depressive-like behaviors in mice. Am J Psychiatry. (2020) 177:974–90. doi: 10.1176/appi.ajp.2020.19090960 PMC7647050 32731813

[182] Xu C Lee SK Zhang D Frenette PS. The gut microbiome regulates psychological-stress-induced inflammation. Immunity. (2020) 53:417–28. doi: 10.1016/j.immuni.2020.06.025 PMC7461158 32735844

[183] Zheng P Li Y Wu J Zhang H Huang Y Tan X. Perturbed microbial ecology in myasthenia gravis: evidence from the gut microbiome and fecal metabolome. Adv Sci. (2019) 6:1901441. doi: 10.1002/advs.201901441 PMC6755540 31559142

[184] Zheng P Zeng B Zhou C Liu M Fang Z Xu X. Gut microbiome remodeling induces depressive-like behaviors through a pathway mediated by the host’s metabolism. Mol Psychiatry. (2016) 21:786–96. doi: 10.1038/mp.2016.44 27067014

[185] Valles-Colomer M Falony G Darzi Y Tigchelaar EF Wang J Tito RY. The neuroactive potential of the human gut microbiota in quality of life and depression. Nat Microbiol. (2019) 4:623–32. doi: 10.1038/s41564-018-0337-x 30718848

[186] Liang S Wu X Hu X Wang T Jin F. Recognizing depression from the microbiota–gut–brain axis. Int J Mol Sci. (2018) 19:1592. doi: 10.3390/ijms19061592 29843470 PMC6032096

[187] Makris AP Karianaki M Tsamis KI Paschou SA. The role of the gut-brain axis in depression: endocrine, neural, and immune pathways. Hormones. (2021) 20:1–12. doi: 10.1007/s42000-020-00236-4 32827123

[188] Yang J Zheng P Li Y Wu J Tan X Zhou J. Landscapes of bacterial and metabolic signatures and their interaction in major depressive disorders. Sci Adv. (2020) 6:eaba8555. doi: 10.1126/sciadv.aba8555 33268363 PMC7710361

[189] Zheng P Yang J Li Y Wu J Liang W Yin B. Gut microbial signatures can discriminate unipolar from bipolar depression. Adv Sci. (2020) 7:1902862. doi: 10.1002/advs.201902862 PMC7140990 32274300

[190] Sanada K Nakajima S Kurokawa S Barceló-Soler A Ikuse D Hirata A. Gut microbiota and major depressive disorder: A systematic review and meta-analysis. J Affect Disord. (2020) 266:1–13. doi: 10.1016/j.jad.2020.01.102 32056863

[191] Ait-Belgnaoui A Colom A Braniste V Ramalho L Marrot A Cartier C. Probiotic gut effect prevents the chronic psychological stress-induced brain activity abnormality in mice. Neurogastroenterol Motil. (2014) 26:510–20. doi: 10.1111/nmo.12295 24372793

[192] Dhakal R Bajpai VK Baek KH. Production of GABA (γ-aminobutyric acid) by microorganisms: A review. Braz J Microbiol. (2012) 43:1230–41. doi: 10.1590/S1517-83822012000400001 PMC3769009 24031948

[193] Chinna Meyyappan A Forth E Wallace CJK Milev R. Effect of fecal microbiota transplant on symptoms of psychiatric disorders: A systematic review. BMC Psychiatry. (2020) 20:299. doi: 10.1186/s12888-020-02654-5 32539741 PMC7294648

[194] Doll JPK Vázquez-Castellanos JF Schaub AC Schweinfurth N Kettelhack C Schneider E. Fecal microbiota transplantation (FMT) as an adjunctive therapy for depression—Case report. Front Psychiatry. (2022) 13. doi: 10.3389/fpsyt.2022.815422 PMC8891755 35250668

[195] Chang M Chang KT Chang F. Just a gut feeling: Faecal microbiota transplant for treatment of depression – A mini-review. J Psychopharmacol. (2024) 38(4):353–61. doi: 10.1177/02698811241240308 38532577

[196] Zhao H Jin K Jiang C Pan F Wu J Luan H. A pilot exploration of multi-omics research of gut microbiome in major depressive disorders. Transl Psychiatry. (2022) 12:8. doi: 10.1038/s41398-021-01769-x 35013099 PMC8748871

[197] Drevets WC Wittenberg GM Bullmore ET Manji HK. Immune targets for therapeutic development in depression: towards precision medicine. Nat Rev Drug Discovery. (2022) 21:224–44. doi: 10.1038/s41573-021-00368-1 PMC8763135 35039676

[198] Schuff N. *In vivo* NMR methods, overview of techniques. In: Encyclopedia of Spectroscopy and Spectrometry. Cambridge, Massachusetts, US: Academic Press (2016).

[199] Arnatkeviciute A Markello RD Fulcher BD Misic B Fornito A. Toward best practices for imaging transcriptomics of the human brain. Biol Psychiatry. (2023) 93:391–404. doi: 10.1016/j.biopsych.2022.10.016 36725139

[200] Fan JW Gu YW Wang DB Liu XF Zhao SW Li X. Transcriptomics and magnetic resonance imaging in major psychiatric disorders. Front Psychiatry. (2023) 14. doi: 10.3389/fpsyt.2023.1185471 PMC10296768 37383618

[201] Sun X Huang W Wang J Xu R Zhang X Zhou J. Cerebral blood flow changes and their genetic mechanisms in major depressive disorder: a combined neuroimaging and transcriptome study. Psychol Med. (2023) 53:1–13. doi: 10.1017/S0033291722003750 36601814

[202] Zhu W Liu F Fu J Qin W Xue K Tang J. Genes associated with spontaneous brain activity changes in clinically different patients with major depressive disorder: A transcription-neuroimaging association study. CNS Neurosci Ther. (2023) 29:3913–24. doi: 10.1111/cns.14311 PMC10651976 37311691

[203] Oh EY Han KM Kim A Kang Y Tae WS Han MR. Integration of whole-exome sequencing and structural neuroimaging analysis in major depressive disorder: a joint study. Transl Psychiatry. (2024) 14:141. doi: 10.1038/s41398-024-02849-4 38461185 PMC10924915

[204] Loscalzo J Kohane I Barabasi AL. Human disease classification in the postgenomic era: A complex systems approach to human pathobiology. Mol Syst Biol. (2007) 3:124. doi: 10.1038/msb4100163 17625512 PMC1948102

[205] Noble D. Neo-Darwinism, the Modern Synthesis and selfish genes: Are they of use in physiology? J Physiol. (2011) 589:1007–15. doi: 10.1113/jphysiol.2010.201384 PMC3060581 21135048

[206] Iyengar R. Complex diseases require complex therapies. EMBO Rep. (2013) 14:1039–42. doi: 10.1038/embor.2013.177 PMC3981088 24232184

[207] Antony PMA Balling R Vlassis N. From systems biology to systems biomedicine. Curr Opin Biotechnol. (2012) 23:604–8. doi: 10.1016/j.copbio.2011.11.009 22119097

[208] Pontrelli G Olufsen MS Ottesen JT. Mathematical methods and models in system biomedicine. Math Biosci. (2014) 257:1. doi: 10.1016/j.mbs.2014.09.012 25443447

[209] Capobianco E Lió P. Advances in translational biomedicine from systems approaches. Front Genet. (2014) 5. doi: 10.3389/fgene.2014.00273 PMC4132370 25177342

[210] Vandereyken K Sifrim A Thienpont B Voet T. Methods and applications for single-cell and spatial multi-omics. Nat Rev Genet. (2023) 24:494–515. doi: 10.1038/s41576-023-00580-2 PMC9979144 36864178

[211] Wassermann AM Lounkine E Glick M. Bioturbo similarity searching: Combining chemical and biological similarity to discover structurally diverse bioactive molecules. J Chem Inf Model. (2013) 53:692–703. doi: 10.1021/ci300607r 23461561

[212] Singh A Shannon CP Gautier B Rohart F Vacher M Tebbutt SJ. DIABLO: An integrative approach for identifying key molecular drivers from multi-omics assays. Bioinformatics. (2019) 35:3055–62. doi: 10.1093/bioinformatics/bty1054 PMC6735831 30657866

[213] Uppal K Ma C Go YM Jones DP. XMWAS: A data-driven integration and differential network analysis tool. Bioinformatics. (2018) 34:701–2. doi: 10.1093/bioinformatics/btx656 PMC5860615 29069296

[214] Nuñez NA Joseph B Pahwa M Kumar R Resendez MG Prokop LJ. Augmentation strategies for treatment resistant major depression: A systematic review and network meta-analysis. J Affect Disord. (2022) 302:385–400. doi: 10.1016/j.jad.2021.12.134 PMC9328668 34986373

[215] Jha MK Mathew SJ. Pharmacotherapies for treatment-resistant depression: how antipsychotics fit in the rapidly evolving therapeutic landscape. Am J Psychiatry. (2023) 180:190–9. doi: 10.1176/appi.ajp.20230025 36855876

[216] Pandarakalam JP. Challenges of treatment-resistant depression. Psychiatr Danub. (2018) 30:273–84. doi: 10.24869/psyd. 30267518

[217] Leonard B Maes M. Mechanistic explanations how cell-mediated immune activation, inflammation and oxidative and nitrosative stress pathways and their sequels and concomitants play a role in the pathophysiology of unipolar depression. Neurosci Biobehav Rev. (2012) 36:764–85. doi: 10.1016/j.neubiorev.2011.12.005 22197082

[218] Mehltretter J Rollins C Benrimoh D Fratila R Perlman K Israel S. Analysis of features selected by a deep learning model for differential treatment selection in depression. Front Artif Intell. (2020) 2. doi: 10.3389/frai.2019.00031 PMC7861264 33733120

[219] Alowais SA Alghamdi SS Alsuhebany N Alqahtani T Alshaya AI Almohareb SN. Revolutionizing healthcare: the role of artificial intelligence in clinical practice. BMC Med Educ. (2023) 23:689. doi: 10.1186/s12909-023-04698-z 37740191 PMC10517477

[220] Hamet P Tremblay J. Artificial intelligence in medicine. Metabolism. (2017) 69:S36–40. doi: 10.1016/j.metabol.2017.01.011 28126242

[221] Wei L Niraula D Gates EDH Fu J Luo Y Nyflot MJ. Artificial intelligence (AI) and machine learning (ML) in precision oncology: a review on enhancing discoverability through multiomics integration. Br J Radiol. (1150) 2023:96. doi: 10.1259/bjr.20230211 PMC10546458 37660402

[222] Fitzpatrick KK Darcy A Vierhile M. Delivering cognitive behavior therapy to young adults with symptoms of depression and anxiety using a fully automated conversational agent (Woebot): A randomized controlled trial. JMIR Ment Heal. (2017) 4:e19. doi: 10.2196/mental.7785 PMC5478797 28588005

[223] Gabbard GO Crisp-Han H. The early career psychiatrist and the psychotherapeutic identity. Acad Psychiatry. (2017) 41:30–4. doi: 10.1007/s40596-016-0627-7 27882522

[224] Gao S Calhoun VD Sui J. Machine learning in major depression: From classification to treatment outcome prediction. CNS Neurosci Ther. (2018) 24:1037–52. doi: 10.1111/cns.13048 PMC6324186 30136381

[225] Vidal-Alaball J Fibla DR Zapata MA Marin-Gomez FX Fernandez OS. Artificial intelligence for the detection of diabetic retinopathy in primary care: Protocol for algorithm development. JMIR Res Protoc. (2019) 8:e12539. doi: 10.2196/12539 30707105 PMC6376335

[226] Cortes-Briones JA Tapia-Rivas NI D’Souza DC Estevez PA. Going deep into schizophrenia with artificial intelligence. Schizophr Res. (2022) 245:122–40. doi: 10.1016/j.schres.2021.05.018 34103242

[227] Kleinerman A Rosenfeld A Benrimoh D Fratila R Armstrong C Mehltretter J. Treatment selection using prototyping in latent-space with application to depression treatment. PloS One. (2021) 16:e0258400. doi: 10.1371/journal.pone.0258400 34767577 PMC8589171

[228] Li Z Lai J Zhang P Ding J Jiang J Liu C. Multi-omics analyses of serum metabolome, gut microbiome and brain function reveal dysregulated microbiota-gut-brain axis in bipolar depression. Mol Psychiatry. (2022) 27:4123–35. doi: 10.1038/s41380-022-01569-9 35444255

[229] Bilello JA. Seeking an objective diagnosis of depression. biomark Med. (2016) 10:861–75. doi: 10.2217/bmm-2016-0076 27415130

[230] Harris MG Kazdin AE Chiu WT Sampson NA Aguilar-Gaxiola S Al-Hamzawi A. Findings from world mental health surveys of the perceived helpfulness of treatment for patients with major depressive disorder. JAMA Psychiatry. (2020) 77:830–41. doi: 10.1001/jamapsychiatry.2020.1107 PMC7240636 32432716

[231] Ali FZ Parsey RV Lin S Schwartz J DeLorenzo C. Circadian rhythm biomarker from wearable device data is related to concurrent antidepressant treatment response. NPJ Digit Med. (2023) 6:81. doi: 10.1038/s41746-023-00827-6 37120493 PMC10148831

[232] Anmella G Corponi F Li BM Mas A Sanabra M Pacchiarotti I. Exploring Digital biomarkers of illness activity in mood episodes: hypotheses generating and model development study. JMIR mHealth uHealth. (2023) 11:e45405. doi: 10.2196/45405 36939345 PMC10196899

[233] Koga N Komatsu Y Shinozaki R Ishida I Shimizu Y Ishimaru S. Simultaneous monitoring of activity and heart rate variability in depressed patients: A pilot study using a wearable monitor for 3 consecutive days. Neuropsychopharmacol Rep. (2022) 42:457–67. doi: 10.1002/npr2.12285 PMC9773773 35906793

[234] Matcham F Leightley D Siddi S Lamers F White KM Annas P. Remote Assessment of Disease and Relapse in Major Depressive Disorder (RADAR-MDD): recruitment, retention, and data availability in a longitudinal remote measurement study. BMC Psychiatry. (2022) 22:136. doi: 10.1186/s12888-022-03753-1 35189842 PMC8860359

[235] Yuan B Li J. The policy effect of the general data protection regulation (GDPR) on the digital public health sector in the european union: An empirical investigation. Int J Environ Res Public Health. (2019) 16:1070. doi: 10.3390/ijerph16061070 30934648 PMC6466053

[236] Schaake M. European Commission’s Artificial Intelligence Act. Stanford, California, US: Stanford University, Human-Centered Artificial Intelligence (2021).

[237] Cohen IG Mello MM. HIPAA and protecting health information in the 21st Century. JAMA - J Am Med Assoc. (2018) 320:231–2. doi: 10.1001/jama.2018.5630 29800120

